# Molecular mechanism of Fe^3+^ binding inhibition to *Vibrio metschnikovii* ferric ion‐binding protein, FbpA, by rosmarinic acid and its hydrolysate, danshensu

**DOI:** 10.1002/pro.4881

**Published:** 2024-02-01

**Authors:** Peng Lu, Jinyan Jiang, Chang Liu, Suguru Okuda, Hideaki Itoh, Ken Okamoto, Michio Suzuki, Koji Nagata

**Affiliations:** ^1^ Department of Applied Biological Chemistry, Graduate School of Agricultural and Life Science The University of Tokyo Tokyo Japan; ^2^ Agricultural Bioinformatics Research Unit, Graduate School of Agricultural and Life Science The University of Tokyo Tokyo Japan; ^3^ Research Center for Food Safety, Graduate School of Agricultural and Life Science The University of Tokyo Tokyo Japan

**Keywords:** crystallography, danshensu, ferric ion, foodborne illnesses, global warming, iron‐binding site, rosmarinic acid

## Abstract

Global warming has increased the growth of pathogenic *Vibrio* bacteria, which can cause foodborne illnesses and death. *Vibrio* bacteria require iron for growth and survival. They utilize a ferric ion‐binding protein (FbpA) to bind and transport Fe^3+^ into the cell. FbpA from *Vibrio metschnikovii* (Vm) is a potential target for inhibiting its growth. Rosmarinic acid (RA) can block the binding of VmFbpA to Fe^3+^; however, the molecular mechanism of Fe^3+^ binding and RA inhibition to VmFbpA is unclear. In this study, we used x‐ray crystallography to determine the Fe^3+^‐binding mode of VmFbpA and the mechanism of RA inhibition. The structures revealed that in the Fe^3+^ bound form, Fe^3+^ was coordinated to VmFbpA by two Tyr residues, two HCO_3_^−^ ions, and two water molecules in a six‐coordinated geometry. In contrast, in the inhibitor bound form, RA was initially bound to VmFbpA following gel filtration purification, but it was hydrolyzed to danshensu (DSS), which occupied the binding site as shown in an electron density map and reverse phase chromatography (RPC) analysis. Both RA and DSS exhibited a stronger binding affinity to VmFbpA, higher Fe^3+^ reduction capacity, and more potent bacteriostatic effect on *V. metschnikovii* compared with caffeic acid (CA), another hydrolysis product of RA. These results provide insight into the mechanism of iron acquisition by *V. metschnikovii* and inhibition by RA and DSS. Our findings offer clues on the development of effective strategies to prevent *Vibrio* infections.

## 1 INTRODUCTION

Rising ocean temperatures have been linked to the proliferation of marine bacteria, including pathogenic and non‐pathogenic *Vibrio* species. In the United States, *Vibrio* species account for an estimated 80,000 illnesses and 100 deaths annually, with the majority being foodborne (Authority (EFSA) EFS et al. 2020; Newton et al. 2012). Notably, infections peak during the warmer months of July and August and are not limited to common strains but also a less common strain (e.g., *Vibrio metschnikovii*), which can cause vibriosis through various exposures (Baker‐Austin et al. 2018; Brumfield et al. 2021).

In July 2021, *V. metschnikovii* was reported to be the dominant strain that caused a high mortality rate in hybrid sturgeon disease in Zhengzhou, Henan Province, China. This suggests that *V. metschnikovii* should also be considered a potential pathogen in the aquaculture industry and highlights the importance of regular monitoring and management practices to prevent outbreaks of vibriosis in aquaculture (Xiao et al. 2022).

Iron is an essential element for almost all living organisms and the *Vibrio* species is no exception. For the growth and survival of *Vibrio* bacteria, iron has a central role in many biological processes, such as DNA replication, metabolism, and energy production. To support the general biological function in bacteria, ~10^−6^ to 10^−7^ molar iron is required for one cell. In contrast, the *Vibrio* species requires iron concentrations between 0.1 μM and 5.0 μM to promote bacterial growth (Payne et al. 2016).

**FIGURE 1:**
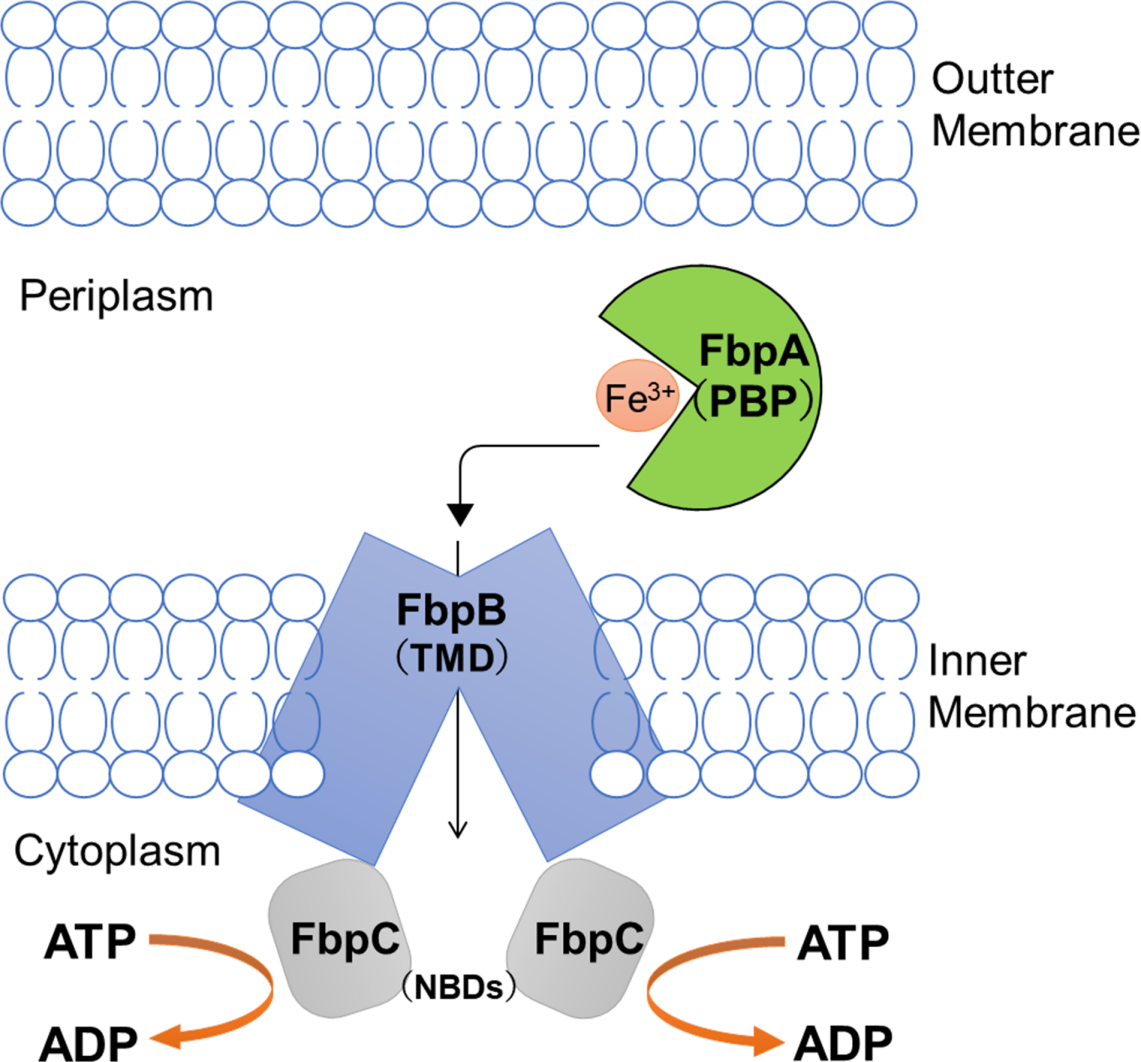
Schematic model of Fe^3+^ transportation by the Fbp system in Gram‐negative bacteria. Fbp, ferric ion‐binding protein; NBD, nucleotide‐binding domain; PBP, periplasmic binding protein; TMD, transmembrane domain.

Most gram‐negative bacteria contain the highly conserved FbpA protein, which is considered bacterial transferrin and binds to free Fe^3+^. It is also known as periplasmic binding protein and belongs to the importer family of the ATP‐binding cassette transporters (Parker Siburt et al. 2012). In addition to FbpA, the protein has two additional subunits. One is a transmembrane domain that spans the inner membrane. It forms a complex with the other nucleotide‐binding domain in the cytoplasm and uses ATP hydrolysis energy to transport Fe^3+^ from the periplasmic side to the cytoplasm. Therefore, FbpA can bind Fe^3+^ in the periplasm of gram‐negative bacteria and transport it intracellularly through a transmembrane channel while consuming ATP during transport (Figure 1) (Lu et al. 2019).

With the rise in multidrug‐resistant bacterial strains, innovative approaches have become essential to inhibit their growth. Our previous investigations have explored the inhibition of iron acquisition in bacteria by targeting the ferric ion‐binding protein (FbpA). Specifically, we suppressed the growth of pathogenic *V. metschnikovii* by inhibiting VmFbpA using naturally occurring food components. This strategy could offer a selective means of bacterial control with fewer side effects on the human body (Lu et al. 2021).

We systematically screened various extracts from spices for VmFbpA inhibition and identified rosmarinic acid (RA) from rosemary. This compound exhibits a stronger binding affinity to VmFbpA than Fe^3+^. This competitive binding suggests that RA effectively inhibits the iron‐binding of VmFbpA, which we have previously shown to significantly restrict the growth of *V. metchnikovii*. Our antibacterial tests further demonstrated that RA at 1000 μM concentration significantly reduced the growth of *V. metchnikovii*. However, notably, RA at this concentration did not affect the growth of *Escherichia coli*, a common indigenous gut bacterium, thus highlighting the selective inhibitory action of RA against *V. metchnikovii* (Lu et al. 2021).

Based on these findings, the present study performed structural analysis of VmFbpA to elucidate the Fe^3+^‐binding mode and unravel the molecular mechanism of RA inhibition. Our work offers novel insights into the iron acquisition process in *V. metchnikovii* and contributes to the development of effective strategies to mitigate the spread of *V. metschnikovii*.

## 2 RESULTS

### 2.1 Structure determination of the Fe^3+^‐ and RA‐ bound VmFbpA

The purification and preparation of Fe^3+^‐ and RA‐treated VmFbpA were shown in Figure S1. It was observed that the apo form of VmFbpA is colorless and transparent. When treated with high concentrations of Fe^3+^, the protein color became brown, whereas RA treatment resulted in a green coloration. These color changes showed that the binding of ligands remained evident even after gel filtration.

Diffraction‐quality crystals of Fe^3+^‐bound VmFbpA were obtained with a reservoir composition of 0.2 M magnesium formate dihydrate and 20% (w/v) PEG 3350, pH 7.5. The best crystal diffracted x‐ray was obtained at a maximum resolution of ~2.15 Å (Figure S2a). Diffraction‐quality crystals of RA‐treated VmFbpA were obtained with a reservoir composition of 1 M (NH_4_)_2_HPO_4_ and 0.1 M imidazole‐HCl (pH 8.0) (Figure S2b). The best crystal diffracted x‐ray was obtained at a maximum resolution of ~2.00 Å. The x‐ray data collection and refinement statistics are summarized in Tables S1 and S2.

**FIGURE 2:**
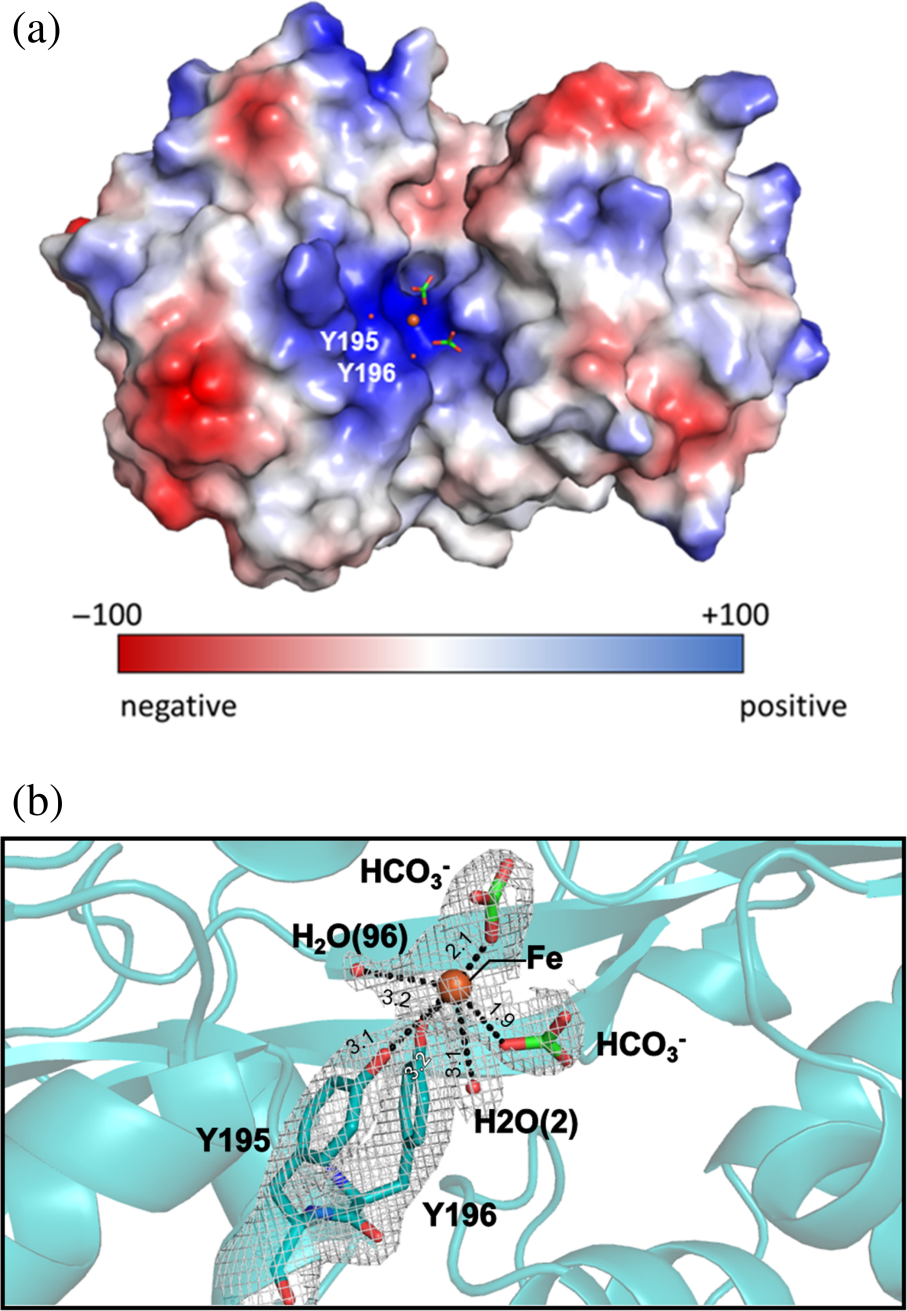
Crystal structure of Fe^3+^ bound VmFbpA. (a) Stick model of Fe^3+^ bound VmFbpA and the 2F_o_‐F_c_ electron density map contoured at 1 σ. (b) The rotation of (a) by 90° along the horizontal axis. (c) The hydrophobicity map of Fe^3+^ bound VmFbpA. (d) The electrostatic potential map of Fe^3+^ bound VmFbpA. (e) The specific Fe^3+^ coordination model for VmFbpA. Fe^3+^ is shown as an orange ball. Two water molecules involved in Fe^3+^ binding are shown as red balls. Two carbonate molecules are shown in green stick models. In the main text, the number in the parentheses following H_2_O represents the number of the water molecule in the PDB file.

For the Fe^3+^‐bound VmFbpA crystal structure, only one protein molecule was present in the asymmetric unit. The Fe^3+^ directly bound to the protein in a six‐coordinated form, which involved two Tyr residues (Tyr195, Tyr196 from VmFbpA), two HCO_3_^−^ ions, and two water molecules (Figure 2). The 2F_O_‐F_C_ map for Fe^3+^ bound VmFbpA at 1, 2, 3, and 4σ was presented in Figure S3a–d.

**FIGURE 3:**
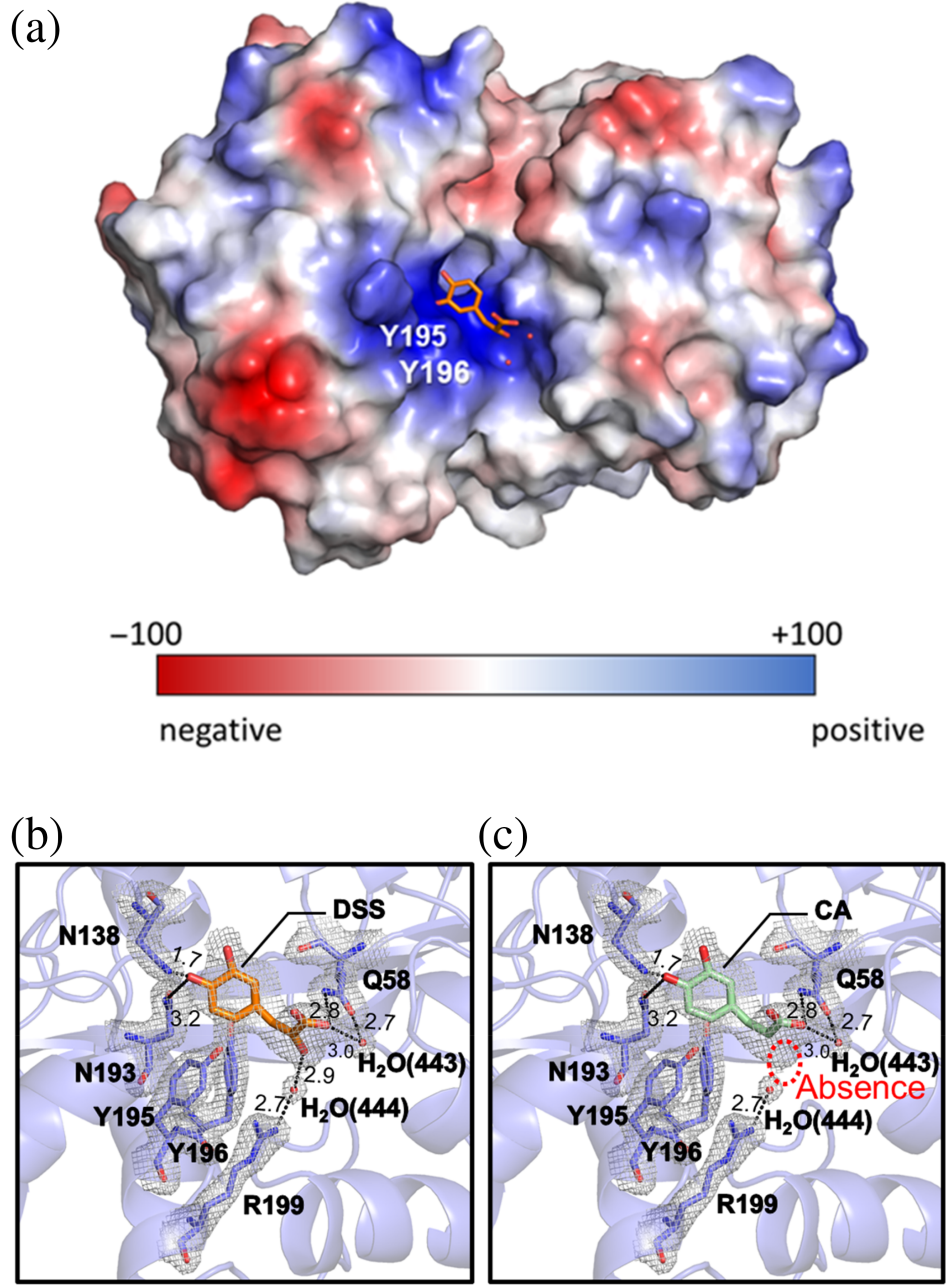
Crystal structure of inhibitor bound VmFbpA. (a) Stick model of inhibitor bound VmFbpA and the 2F_o_‐F_c_ electron density map contoured at 1 σ. (b) The rotation of (a) by 90° along the horizontal axis. (c) The hydrophobicity map of inhibitor bound VmFbpA. (d) The electrostatic potential map of inhibitor bound VmFbpA. (e) The specific interaction between DSS and VmFbpA. (f) The specific interaction between CA and VmFbpA. Two water molecules involved in the interaction are shown as red balls. DSS is shown in orange stick models. CA is shown in light green stick models. In the main text, the number in the parentheses following H_2_O represents the number of the water molecule in the PDB file.

For the RA‐treated VmFbpA crystal structure, only one protein molecule was present in the asymmetric unit. When the electron density map was contoured at ~1 sigma, the electron density from the ligand was clearly observed at the Fe^3+^‐binding site, but the size was only half compared with that of the RA (Figure 3). Both the left half (CA) and right half (DSS) of RA (Figure S4) fit well with the electron density map (Figure 3b,c). When fit with DSS, five hydrogen bonds were formed by the interaction, which included Gln58, Asn138, Asn193, and two water molecules (Figure 3b). When fit with CA, four hydrogen bonds were formed by the interaction, which included Gln58, Asn138, Asn193, and one water molecule (Figure 3c). F_O_‐F_C_ maps for DSS bound VmFbpA (R factor: 0.172) and CA‐bound VmFbpA (R factor: 0.175) were presented in Figure S3e–l.

### 2.2 Ligand determination

**FIGURE 4:**
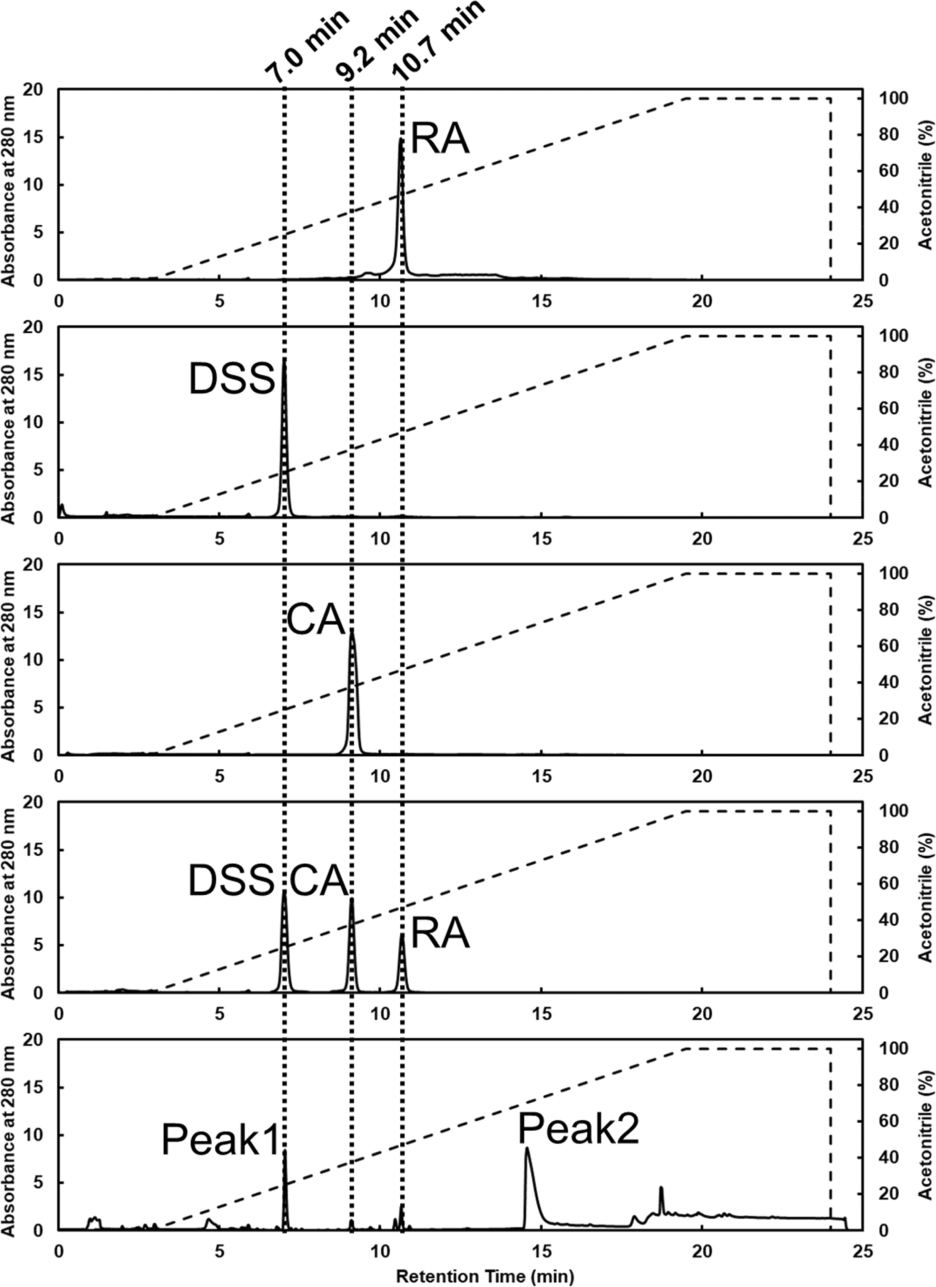
RPC analysis. The chromatographic curves from top to bottom for RA, DSS, CA, and the mixture (RA, DSS, and CA), and VmFbpA crystals prepared by the addition of RA. The UV absorbance of the chromatographic curves of the standard samples were standardized. The dotted line represents the gradient for acetonitrile.

**FIGURE 5:**
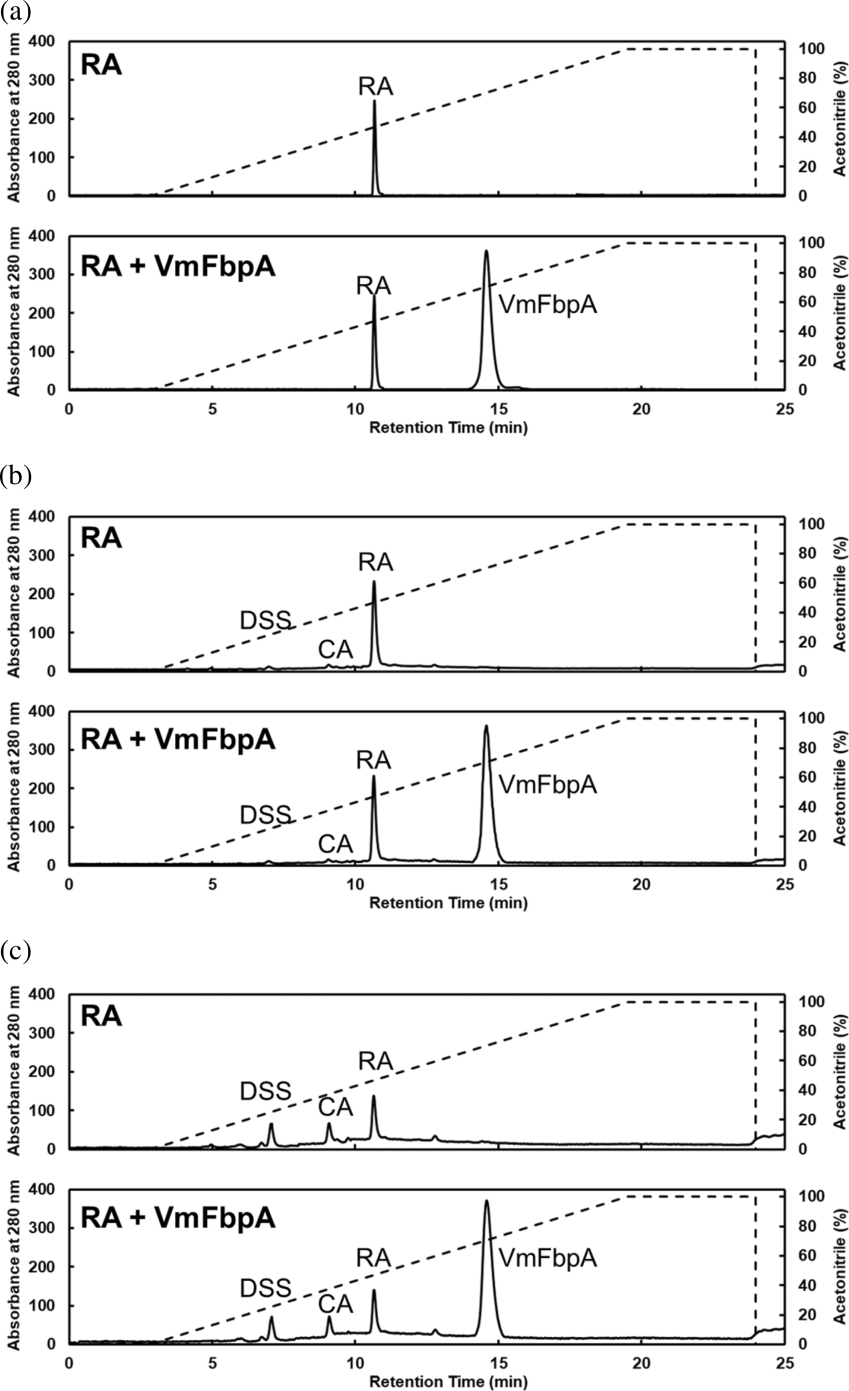
Reverse phase chromatography (RPC) analysis. (a) Rosmarinic acid (RA) in the absence and presence of VmFbpA under 20 mM Tris–HCl, pH 8.0, and 100 mM NaCl. (b) The same samples from (a), but stored at 20°C for 1 week. (c) The same samples from (a) but stored at 20°C for 2 weeks.

To determine the ligand bound to VmFbpA in the RA‐treated samples, the crystals were collected, dissolved in solvent buffer A (1% acetonitrile, 0.1% TFA, and 98.9% Milli‐Q), and analyzed by RPC. Pure RA, DSS, and CA were used as the standard samples. As shown in Figures 4 and 5, the RPC results indicated that the retention volumes of RA, DSS, and CA were 7.0, 9.2, and 10.7 min, respectively. The chromatographic pattern of RA‐treated VmFbpA crystal samples presented two significant peaks (Figure 4). Peak 1 was a sharp peak with a retention time identical to that of DSS. Peak 2 appeared as a broad peak in the RPC chromatogram with a retention time of ~14.5 min, which suggested that it corresponded to the VmFbpA protein peak.

**FIGURE 6:**
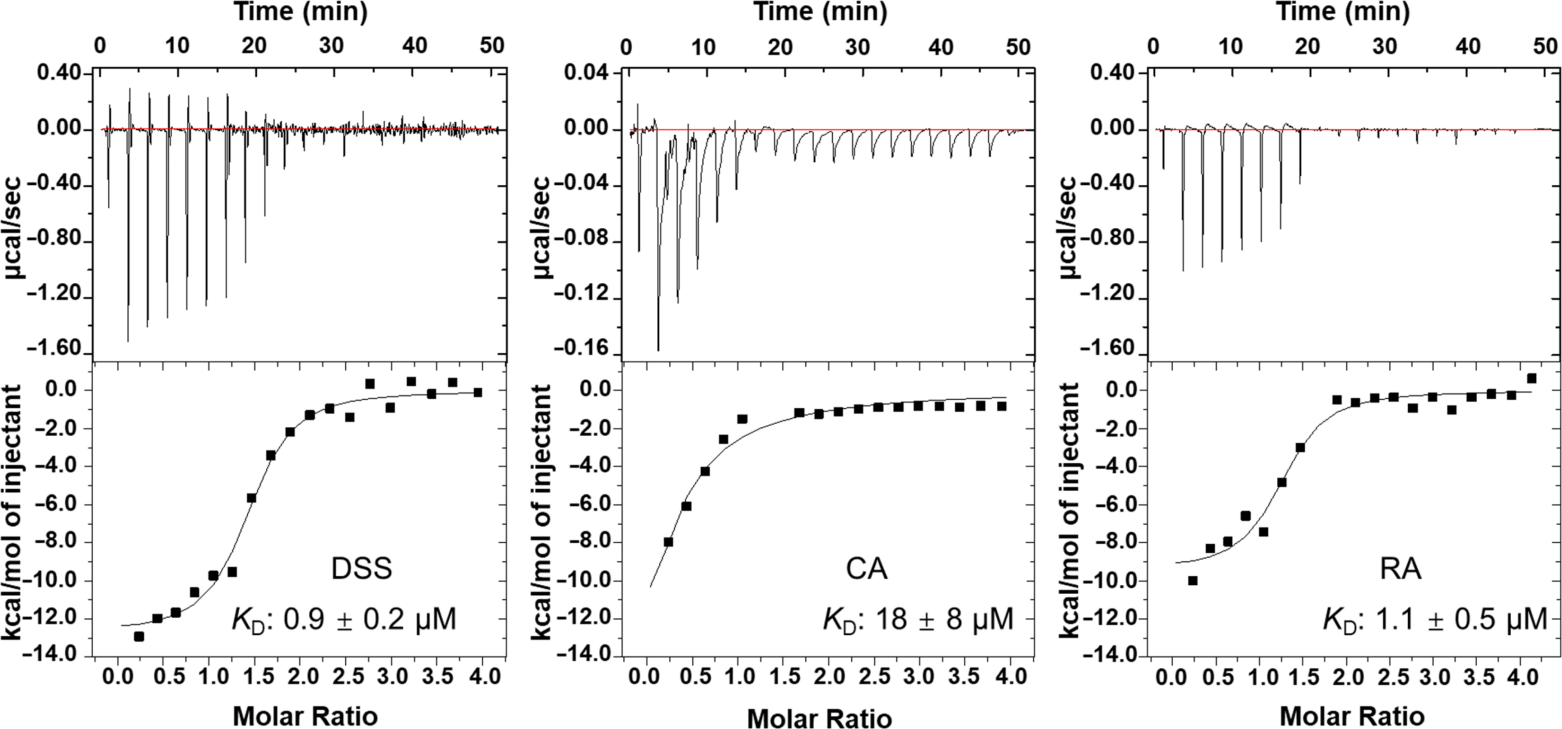
ITC analysis of RA, DSS, and CA binding to VmFbpA at 298 K in 20 mM HEPES, pH 8.0, and 50 mM NaCl.

Furthermore, the binding affinities of RA, DSS, and CA for VmFbpA were measured by isothermal titration calorimetry (ITC) and the corresponding *K*_D_ values were 1.1 ± 0.5 μM, 0.9 ± 0.2 μM, and 18 ± 8 μM, respectively (Figure 6).

### 2.3 Biochemical activities of RA, DSS, and CA

**FIGURE 7:**
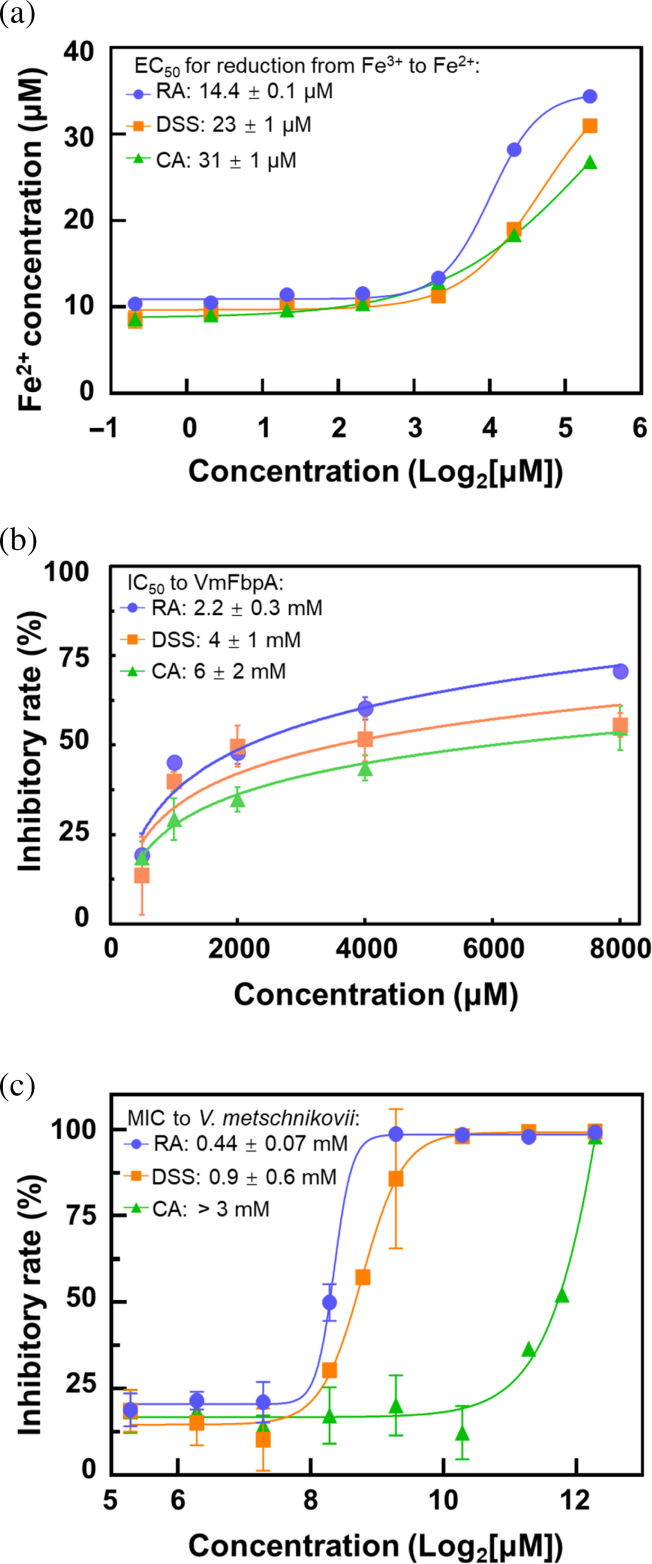
Activity comparison of rosmarinic acid (RA), danshensu (DSS), and caffeic acid (CA). (a) Comparison of the abilities of RA, DSS, and CA for reducing Fe^3+^ to Fe^2+^ (based on the data presented in Figure S4A). (b) Comparison of the inhibitory effects of RA, DSS, and CA on Fe^3+^ binding to VmFbpA (based on the data presented in Figure S4B). (c) Comparison of the inhibitory effects of RA, DSS, and CA on the growth of *V. metschnikovii* (based on the data presented in Figure S5).

The Fe^3+^ reduction capacities of RA, DSS, and CA were evaluated by combining 40 μM Fe^3+^ with various concentrations of RA, DSS, and CA. RA exhibited the highest reduction ability with an EC_50_ value of 14.4 ± 0.1 μM. The EC_50_ values for the hydrolyzed products, DSS and CA, were 23 ± 1 μM and 31 ± 1 μM, respectively (Figure 7a).

To determine the inhibitory activities of RA, DSS, and CA on Fe^3+^ binding to VmFbpA, a 6 × His‐based pull‐down assay was performed. The results indicated that RA had the highest inhibitory activity with an IC_50_ value of 2.2 ± 0.3 mM, followed by DSS and CA with IC_50_ values of 4 ± 1 mM and 6 ± 2 mM, respectively (Figure 7b).

To assess the bacteriostatic effects of RA, DSS, and CA on *V. metschnikovii*, the bacterium was cultured in nutrient broth (NB) medium supplemented with a range of RA, DSS, and CA concentrations. minimum inhibitory concentration (MIC) values were calculated after a 20 h culture. The results indicated that RA had the strongest bacteriostatic effect with an MIC value of 0.44 ± 0.07 mM, followed by DSS with a MIC value of 0.9 ± 0.6 mM. CA exhibited the lowest bacteriostatic effect with a MIC value greater than 3 mM (Figure 7c).

## 3 DISCUSSION

### 3.1 Interaction between Fe^3+^ and VmFbpA

The electrostatic potential map of VmFbpA revealed that the iron‐binding sites tend to have a positive electrostatic potential (Figures 2a and 3a). This creates a repulsive force when Fe^3+^ gets close to it. To neutralize the repulsive force, negatively charged ions, which are considered synergistic anions (Parker Siburt et al. 2012), are needed during Fe^3+^ binding. The pH of the bacterial periplasm is slightly lower than that of the outside environment. In many cases, the periplasmic pH (Stock et al. 1977; O'Keefe and Collier 1989; Dhungana et al. 2003; Bruscella et al. 2005; Wilks and Slonczewski 2007; Heymann et al. 2010) is ~6.5. This pH level enables various anions to serve as synergistic anions for FbpAs. The synergistic anions for FbpA include SO_4_^2−^, HPO_4_^2−^, HAsO_4_^2−^, citrate^3−^, NTA^3−^, HP_2_O_7_^3−^, oxalate^2−^, and HCO_3_^−^/CO_3_^2−^ ions (Parker Siburt et al. 2012). These synergistic anions (Bruns et al. 1997; Bruns et al. 2001; Dhungana et al. 2003; Heymann et al. 2007; Perico 2012) are all attracted toward the positively charged Fe^3+^‐binding site to stabilize Fe^3+^.

**FIGURE 8:**
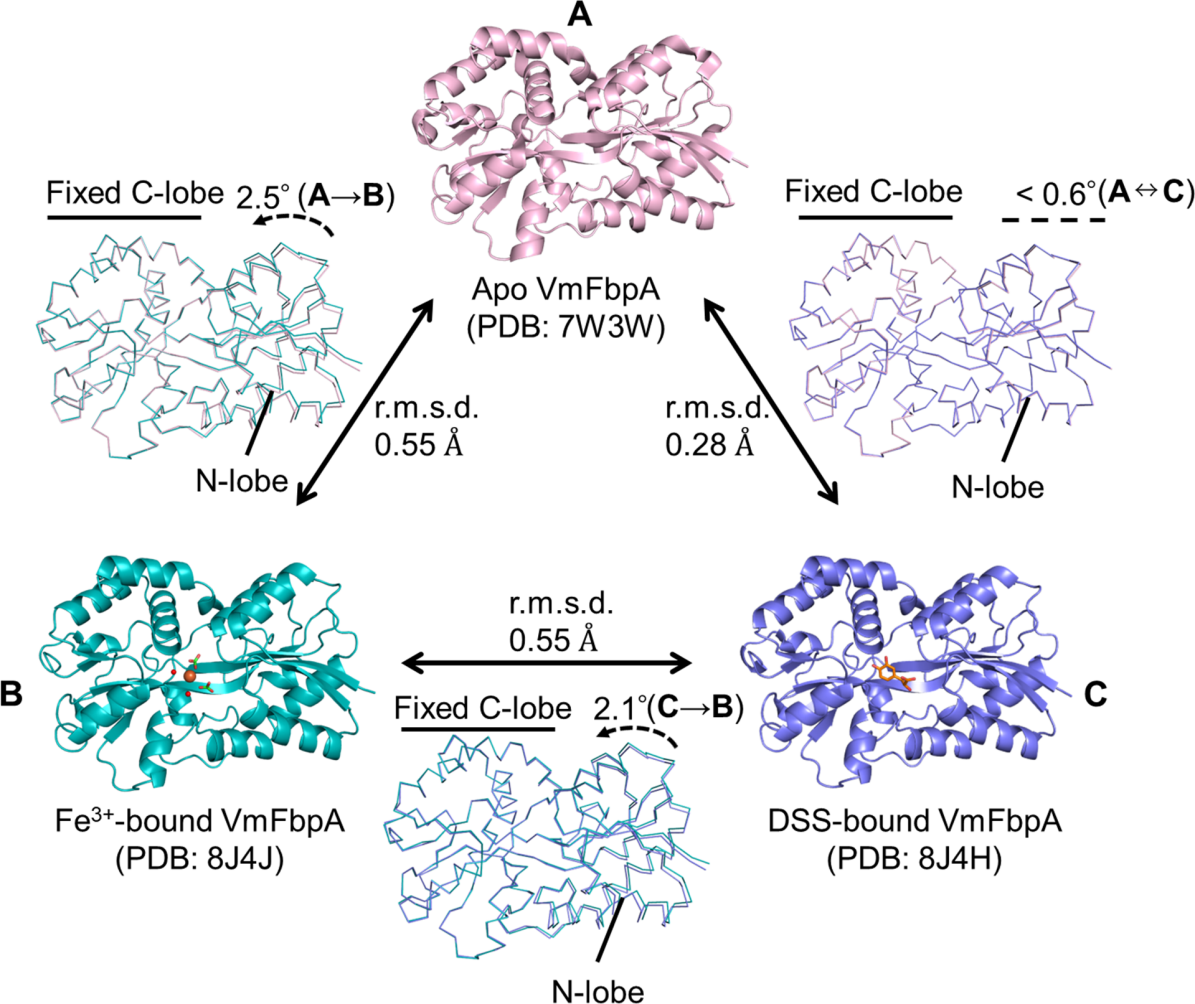
Conformational changes of apo VmFbpA (a), Fe^3+^ bound VmFbpA (b), and inhibitor bound VmFbpA (c).

**FIGURE 9:**
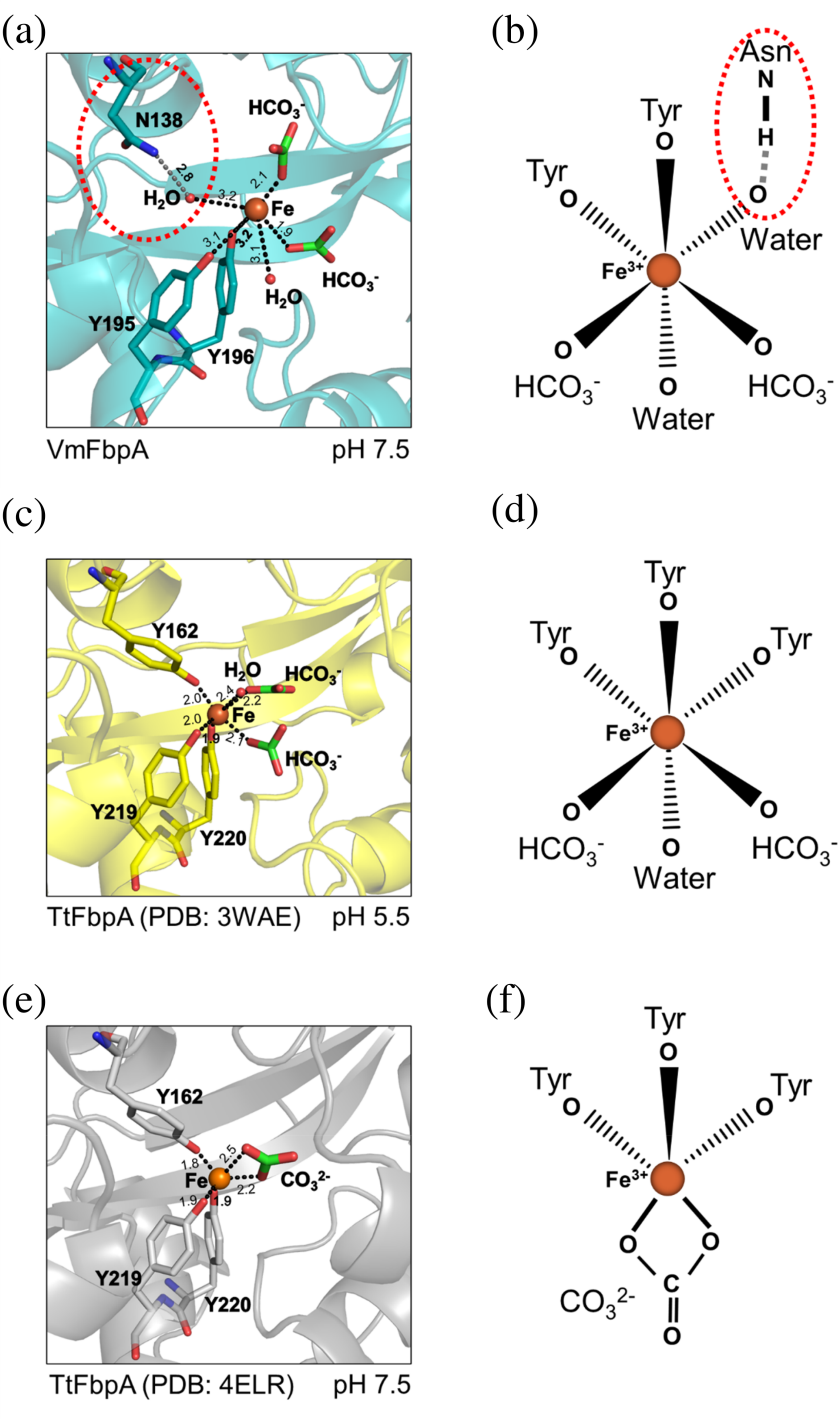
Comparison of Fe^3+^ bound states of VmFbpA and TtFbpA. (a, b) represent the interaction of VmFbpA with Fe^3+^ and its coordination model, respectively. (c, d) represent the interaction of TtFbpA with Fe^3+^ under weakly acidic conditions and its coordination model, respectively. (e, f) represent the interaction of TtFbpA with Fe^3+^ under weakly alkaline conditions and its coordination model, respectively. In the main text, the number in the parentheses following H_2_O represents the number of the water molecule in the PDB file.

In this study, crystallizations of VmFbpA along with a 10‐fold molar quantity of Fe^3+^ and without synergistic anions were also performed; however, none of these contained bound Fe^3+^. When crystallized with a 10‐fold molar quantity of Fe^3+^ and synergistic anions (HCO_3_^−^), the complex of Fe^3+^ bound to VmFbpA was observed. A comparison between Fe^3+^ bound to VmFbpA and apo VmFbpA (PDB: 7W3W) showed that the protein became more compact after Fe^3+^ binding (Figure 8). After fixing the C‐lobe of the protein, a rotation of the N‐lobe by ~2.5° was observed in the Fe^3+^‐bound VmFbpA structure, which indicated that such a conformational change is necessary for VmFbpA to interact and stabilize the ligands (Figure 8). Furthermore, based on the electron density map, Fe^3+^ was bound to VmFbpA in a six‐coordinated mode (Figure 2b), which involved two Tyr residues (Tyr195, Tyr196), two water molecules and two HCO_3_^−^ (Figures 2b and 9a,b).

A BLAST search identified TtFbpA from *Thermus thermophilus* HB8 as the protein with the highest sequence similarity to VmFbpA; a sequence identity of ~42%. Therefore, the Fe^3+^ coordination modes between VmFbpA (resolution: ~2.15 Å) and TtFbpA were compared (Figure 9). TtFbpA can coordinate with Fe^3+^ in two different forms using HCO_3_^−^ or CO_3_^2−^, which form a six‐coordinated mode under acidic conditions (pH 5.5; resolution: ~1.70 Å; PDB: 3WAE) (Figure 9c,d) (Wang et al. 2014) and a five‐coordinated mode under alkaline conditions (pH 7.5; resolution: ~2.50 Å; PDB: 4ELR) (Figure 9e,f) (Wang et al. 2011). Based on our previous report, the six‐coordinated form of TtFbpA corresponds to an open holo‐state, whereas the five‐coordinated form represents the closed holo‐state. The closed holo‐state is a strong Fe^3+^‐binding state. The N‐lobe of TtFbpA rotates by ~16° when TtFbpA changes from an open state to a closed state (Lu et al. 2019). However, in VmFbpA, despite binding with Fe^3+^ at pH 7.5, the conformational change of the N‐lobe in VmFbpA was only ~2.5° and thus remained in a partially open state. Therefore, the conformation and coordination mode of VmFbpA was more similar to the six‐coordinated mode of TtFbpA (Wang et al. 2014) (Figure 9). This indicates that VmFbpA is not in a tightly bound state when bound to Fe^3+^, which makes the release of Fe^3+^ relatively easy upon contact with the transmembrane channel; FbpB, thereby facilitates the cytoplasmic transport of iron. However, this non‐tightly‐bound state of Fe^3+^ binding also implies a higher susceptibility to external inhibitors, such as DSS.

Compared with TtFbpA, which has three Tyr residues (Tyr162, Tyr219, and Tyr220) to coordinate Fe^3+^ (Figure 9c,d), VmFbpA only has two Tyr residues. The corresponding place where there should have been a third Tyr was replaced by Asn (Figure 9a). This leads to a decrease in the ability of VmFbpA to bind to Fe^3+^, which may explain the longer distances observed between Tyr195 and Tyr196 of VmFbpA and Fe^3+^ compared with the distance between Tyr219 and Tyr220 of TtFbpA and Fe^3+^. These results are consistent with the apparent *K*_D_ values previously reported using ITC. The apparent *K*_D_ value for the binding of Fe^3+^ to TtFbpA is ~0.3 μM (Lu et al. 2019), whereas the apparent *K*_D_ value for the binding of Fe^3+^ to VmFbpA is ~17 μM (Lu et al. 2021), indicating that the binding of Fe^3+^ to VmFbpA is weaker compared with that of TtFbpA. Besides, to compensate for the Fe^3+^ coordination by the third tyrosine residue (Tyr162) in TtFbpA, a water molecule O(96) is present between the Fe^3+^ and Asn138 in VmFbpA. This water molecule O(96) is located in close proximity to both Fe^3+^ and N^δ2^ of Asn138, with distances of 3.0 Å and 2.9 Å, respectively. This indicates that the side chain amine of Asn138 (N^δ2^) forms a hydrogen bond with a water molecule O(96), which in turn participates in the coordination of the Fe^3+^. This interaction resulted in the stabilization of the entire six‐coordinated mode of the Fe^3+^.

### 3.2 Interaction between inhibitor and VmFbpA

During the preparation and crystallization of the VmFbpA in a complex with RA, RA was added at a 10‐fold higher molar amount than that of VmFbpA. However, in the crystal structure, only a partial electron density of RA was observed at the Fe^3+^‐binding site. Two possible scenarios may account for this phenomenon: (1) RA was in a flexible state with high structural freedom when binding to VmFbpA or (2) RA underwent degradation and one of the degradation products bound to VmFbpA. To determine which of these occurred, RPC analysis was performed on the crystallized complex. The RPC results indicated that the binding with VmFbpA was a degradation product of RA, DSS (Figure 4).

In plants of the Lamiaceae family, the biosynthesis of RA is catalyzed by rosmarinate synthase from Caffeoyl‐CoA and DSS (Levsh et al. 2019) and is created by an ester bond between CA and DSS (Figure S4). The ester bond in RA may be hydrolyzed and degraded under various conditions (Bel‐Rhlid et al. 2009; Liang et al. 2020; Papaemmanouil et al. 2020). Sik et al. (2021) reported that hydrolysis could occur in both the tincture (ethanol) and aqueous solutions of RA during storage, which thus produces CA and DSS. After 3 months of storage, the hydrolysis rate of the RA tincture (ethanol) solution was 10%–30%, whereas that of the RA aqueous solution (Sik et al. 2021) exceeded 10%. These results are consistent with those obtained in our study. To simulate the degradation status of RA under our experimental conditions, RA was dissolved in gel filtration buffer (20 mM Tris–HCl, pH 8.0, and 100 mM NaCl), and a group with VmFbpA was added (molar concentration ratio, 1:1) as a comparison. The degradation rate of RA did not change significantly in the presence of VmFbpA. However, after storing at 20°C for 1 week, a 5% degradation of RA was observed. After 2 weeks of storage, the degradation rate reached ~30% (Figure 5). During crystallization, the time required for VmFbpA and RA to form crystals exceeded 4 months, and some samples required 6 months. Therefore, despite RA and VmFbpA being used for co‐crystallization, only the degradation product of RA was observed.

In the inhibitor bound crystal structure, both CA and DSS fit the electron density well (Figure 3). Interestingly, based on the RPC results, DSS was the dominant ligand bound to VmFbpA (Figure 4). Compared with Fe^3+^‐bound VmFbpA, DSS‐bound VmFbpA underwent a 2.1° rotation in its N‐lobe; however, compared with apo VmFbpA, the protein did not undergo any significant conformational changes (Figure 8). Therefore, the overall conformation of apo VmFbpA was similar to that of DSS‐bound VmFbpA, but the protein bound with Fe^3+^ was more compact compared with apo VmFbpA and DSS‐bound VmFbpA (Figure 8). In the ligand binding region, DSS bound to VmFbpA through hydrophobic interactions and hydrogen bonds (Figure 2a,b). Gln58, Asn138, and Asn193 directly interacted with DSS through hydrogen bonding, including the O^10^ of DSS–N^ε2^ of Gln58, O of H_2_O (443)–O^ε1^ of Gln58, O of H_2_O (443)–O^10^ of DSS, O^13^ of DSS–N^δ2^ of Asn138, and O^13^ of DSS–N^δ2^ of Asn193 (Figure 2b).

The number in parentheses after H_2_O indicates the number of water molecules in the PDB file (PDB ID: 8J4H). Interestingly, Arg199 did not directly interact with DSS, but instead, interacted with one hydroxyl group (O^14^) of DSS through a H_2_O (444). O of H_2_O (444) formed hydrogen bonds with both N^η2^ of Arg199 and O^14^ of DSS, thereby stabilizing the binding of DSS to VmFbpA (Figures 3b and S4). Based on the chemical structure of CA, however, this hydrogen bond (O of H_2_O (444)–O^14^ of DSS) was absent (Figure 3c). This may be the reason that the affinity of CA binding to VmFbpA is lower than DSS. This result is consistent with our ITC results, which showed that the apparent *K*_D_ of DSS bound to VmFbpA at pH 8.0 was ~0.9 μM, which was significantly lower compared with that of CA (~18 μM).

**FIGURE 10:**
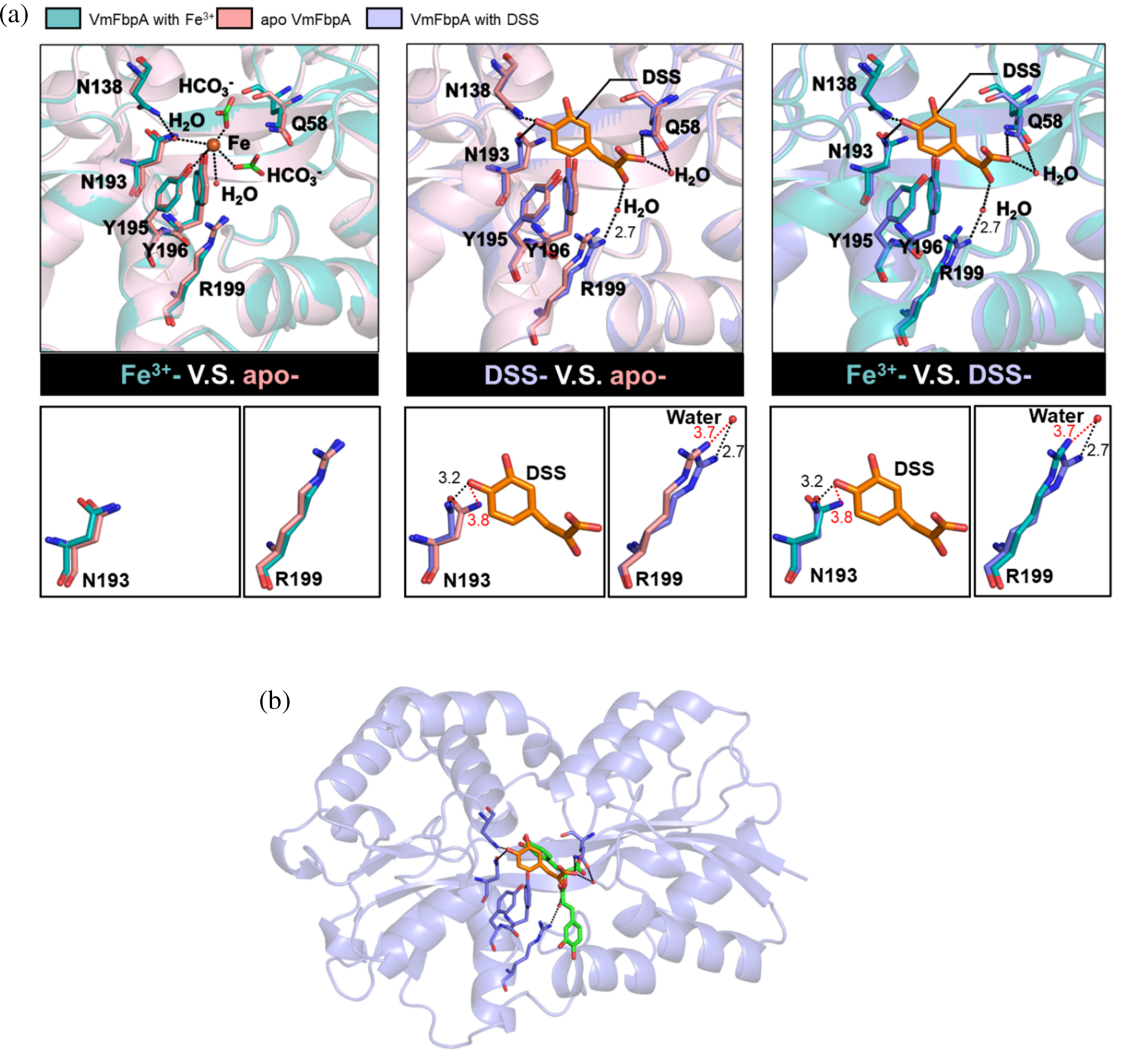
(a) Conformational changes in Fe^3+^‐binding sites of Apo VmFbpA, Fe^3+^‐bound VmFbpA, and danshensu (DSS)‐bound VmFbpA. In the main text, the number in the parentheses following H_2_O represents the number of the water molecule in the PDB file. (b) Comparison between DSS‐bound VmFbpA (crystal structure) and rosmarinic acid (RA)‐bound VmFbpA (docking simulation model). VmFbpA is indicated in purple, DSS in orange, and RA in green.

When comparing DSS‐bound VmFbpA to apo and Fe^3+^‐bound VmFbpA, a change in the position of the side chain of Arg199 and Asn193 was caused by the binding of DSS as an inhibitor (Figure 10a). First, it was clearly observed that there was a shift (from 3.2 Å to 2.7 Å) in the position of N^η2^ of Arg199 toward the O of H_2_O (444), which stabilized DSS binding. In apo VmFbpA, the position of the side chain of Arg199 was similar to that in Fe^3+^‐bound VmFbpA. Second, a shift (from 3.8 Å to 3.3 Å) in the position of N^δ2^ of Asn193 toward the O^13^ of DSS was also observed. In the natural state (apo VmFbpA and Fe^3+^‐VmFbpA), the distance between N^δ2^ of Asn193 and O^13^ of DSS is ~3.8 Å, and the angle for the hydrogen bond is ~79°–90°. This distance and angle are not appropriate to maintain the formation of a hydrogen bond. As illustrated in Figure 10a, when DSS binds, DSS sterically clashes with the Asn193 in the apo conformation, forcing it into a new conformation. Therefore, we believe this steric clash is the driving force for the side chain movement of Asn193.

Moreover, we used a previously reported docking simulation model of RA and VmFbpA with the highest docking score (Bruscella et al. 2005) for comparison with the crystal structure of DSS‐bound VmFbpA. As shown in Figure 10b, the DSS segment of the RA model closely aligned with the position of DSS in the DSS‐bound crystal structure. This indicates a promising starting point for the design of other new inhibitors, and it affirms the reliability and hints at the potential efficacy of the simulation model of compounds with structures analogous to those of RA and DSS.

### 3.3 Comparison of the biochemical activities of RA, DSS, and CA

Although the binding ability of CA to VmFbpA was weaker compared with that of DSS and RA, its inhibitory effect on VmFbpA was comparable to that of DSS and RA (Figure 7b). There was no order of magnitude difference in the binding affinity (Figure 6). This is because the release of Fe^3+^ from VmFbpA may be determined by two factors: (1) competitive binding of inhibitors to the Fe^3+^‐binding site of FbpA, and (2) a reduction of Fe^3+^ to Fe^2+^, which weakens its affinity to FbpA by ~12 orders of magnitude (Dhungana et al. 2003). Although RA, DSS, and CA exhibited differences in their binding abilities to VmFbpA, they had similar abilities in reducing Fe^3+^ to Fe^2+^ (Figure 7a). The reductive ability of Fe^3+^ was evaluated due to the presence of the catechol group in RA, DSS, and CA. This functional group possesses significant reduction properties, allowing it to serve as an electron donor in the reaction. Notably, the number of catechol groups in RA is double than those in DSS and CA, theoretically endowing it with double the reduction ability of the two, which was also consistent with the experimental results (Figure 7a). Based on the EC_50_, although RA had a higher reduction ability compared with DSS, and DSS had a slightly higher reduction ability compared with CA, but there was no order of magnitude difference (Figure 7a). Thus, this could be the reason that no significant difference in their IC_50_ values (Figure 7b) for VmFbpA inhibition was evident.

As inhibitors and bacteriostatic agents, the binding affinities of RA, DSS, and CA to VmFbpA are consistent with their bacteriostatic effects on *V. metschnikovii*. When added to the medium for co‐cultivation, RA, which contains chemical structures of both DSS and CA, exhibited the greatest bacteriostatic effect on the growth of *V. metschnikovii* (MIC was about 0.44 mM) followed by DSS (MIC was about 0.9 mM), whereas the MIC of CA was higher than 3 mM (Figure 7c). This demonstrates that RA and DSS have great potential for use in the aquaculture industry as bacteriostatic agents for inhibiting *V. metschnikovii*.

## 4 CONCLUSIONS

In summary, VmFbpA was purified and crystallized in an Fe^3+^ and inhibitor bound form. In the Fe^3+^ bound form, Fe^3+^ was coordinated to VmFbpA in a six‐coordinated form, involving two Tyr residues, two HCO_3_^−^ ions, and two water molecules. In the inhibitor bound form, RA was bound to VmFbpA after gel filtration purification, but the electron density map and RPC analysis revealed that only half of the molecule (DSS) was present at the binding site. RA and DSS had a stronger binding affinity to VmFbpA, higher Fe^3+^ reduction capacity, and more potent bacteriostatic effects on *V. metschnikovii* compared with CA. These results provide insight into the mechanism of iron acquisition by *V. metschnikovii* and the inhibition by RA and DSS, which suggests a potential role for RA and DSS in inhibiting bacterial growth.

## 5 MATERIALS AND METHODS

### 5.1 Expression and purification of VmFbpA

The gene encoding VmFbpA (WP_004394209.1) was cloned into the pET‐28a vector and overexpressed in *E. coli* BL21 (DE3) after removing the signal peptide at the N‐terminus. A large‐scale culture was prepared in 2 L of LB medium supplemented with IPTG (final concentration of 0.001 mM) at 30°C and an OD_600_ of ~0.6. After induction, the cells were harvested by centrifugation at 4000 × g for 20 min at 4°C and resuspended in lysis buffer (20 mM Tris–HCl, pH 8.0, 100 mM NaCl, 0.50 mg/mL lysozyme, and 1.2 U/mL DNase I). Sonication was done 70% amplitude and 0.5 s on/off for 7 min to disrupt the cells and the resulting lysate was centrifuged at 40,000 × g for 30 min at 4°C. The supernatant was loaded onto a 2 mL Ni‐nitrilotriacetic acid (Ni‐NTA) column and sequentially washed and eluted with binding buffer and elution buffers 1–5, which contained increasing concentrations of imidazole (from 10 mM to 250 mM). The fractions containing 6 × His‐TEV protease‐VmFbpA were collected based on sodium dodecyl sulfate‐polyacrylamide gel electrophoresis (SDS‐PAGE) analysis and dialyzed overnight against dialysis buffer (20 mM pH 8.0 Tris–HCl, 100 mM NaCl) to decrease the imidazole concentration. The 6 × His‐tag was cleaved by adding TEV protease (10 units/mg protein). The reaction mixture was reloaded onto the Ni‐NTA column, and the eluent contained primarily 6 × His‐tag free VmFbpA, which was collected and concentrated to ~15 mg/mL using a Vivaspin 20 (MWCO 30 K, Sartorius). The sample was then subjected to final purification by loading onto a Superdex 200 Increase 10/300 GL column using gel filtration buffer (20 mM pH 8.0 Tris–HCl and 100 mM NaCl) and an ÄKTA purifier (GE Healthcare).

### 5.2 Preparation of Fe^3+^‐ and RA‐ bound VmFbpA

To form apo VmFbpA, 10 mM EDTA was added, and the resulting mixture was subjected to gel filtration. For the generation of Fe^3+^‐bound VmFbpA, the apo protein was incubated at 4°C for 20 h with a 10‐fold (molar) amount of sodium carbonate (Na_2_CO_3_) and ferric chloride hexahydrate (FeCl_3_·6H2O). Similarly, to generate RA‐treated VmFbpA, the apo protein was incubated at 4°C for 20 h with a 10‐fold amount of RA. The samples were purified through a Superdex 200 Increase 10/300 GL column using gel filtration buffer (20 mM Tris–HCl, pH 8.0, and 100 mM NaCl).

### 5.3 Crystallization of Fe^3+^‐ and RA‐treated VmFbpA

Fe^3+^‐bound VmFbpA was concentrated to 7.6 mg/mL using Vivaspin 20 (MWCO 30 K, Sartorius) and crystallized by the sitting‐drop vapor diffusion method in 0.2 M magnesium formate dihydrate and 20% (w/v) PEG 3350 at pH 7.5. RA‐treated VmFbpA was concentrated to 15 mg/mL using Vivaspin 20 (MWCO 30 K, Sartorius) and crystallized by the sitting‐drop vapor diffusion method in 1 M (NH_4_)_2_HPO_4_ and 0.1 M imidazole‐HCl (pH 8.0). The crystals of Fe^3+^‐bound VmFbpA formed after a 1‐week incubation at 20°C grew to a maximum size in 2 weeks, whereas the crystals of VmFbpA supplemented with RA formed after a 3‐month incubation at 20°C and grew to maximum size over 4–6 months.

### 5.4 X‐Ray diffraction data collection and structure determination

X‐ray diffraction experiments were conducted on VmFbpA crystals at beamline BL44XU at SPring‐8 (Hyogo, Japan) and AR‐NE3A at the Photon Factory (Ibaraki, Japan). The crystals were cooled using a stream of cold nitrogen‐gas and cryoprotected in respective reservoirs supplemented with 20% (v/v) glycerol. The final data set for Fe^3+^‐bound VmFbpA crystal was collected at BL44XU at SPring‐8 with a wavelength of 0.899995 Å, a crystal‐to‐detector distance of 220 mm, an oscillation angle of 0.1°, and an exposure time of 0.1 s per image using an EIGER X 16 M detector. The final data set for VmFbpA supplemented with RA was collected at AR‐NE3A at the Photon Factory with a wavelength of 1.00000 Å, a crystal‐to‐detector distance of 201 mm, an oscillation angle of 1°, and an exposure time of 1 s per image using a PILATUS 2 M‐F detector.

The XDS program (Kabsch 2010) was used to perform indexing, integration, and scaling of the x‐ray diffraction data for the VmFbpA complexes. Structural models of these complexes were generated using Morlep (Vagin and Teplyakov 1997) through molecular replacement, with the crystal structures of apo VmFbpA (PDB^9^ entry: 7W3W) serving as an initial model. Further refinement and model building were performed using REFMAC5 (Murshudov et al. 2011), Coot (Emsley and Cowtan 2004), and Phenix (Afonine et al. 2012). The tertiary structure was visualized using PyMOL (Anon Pymol: an open‐source molecular graphics tool – ScienceOpen n.d.).

### 5.5 RPC analysis

The ligands in the crystal structure obtained by RA supplementation were identified by RPC using an ÄKTA purifier. A total of ~30 crystals were picked up in a mounting loop and the reservoir solutions were carefully removed using filter paper. The crystals were dissolved in solvent A1 (1% acetonitrile, 0.1% TFA, and 98.9% Milli‐Q) at a final volume of 1.0 mL and injected into a RESOURCE RPC column (Cytiva) with a column volume (CV) of 3 mL. Solvent A and solvent B (99% acetonitrile, 0.08% TFA and 0.92% Milli‐Q) were used as the mobile phase. The gradient elution program was as follows: 0–2 CV, 0% B; 2–13 CV, 0%–100% B; 13–16 CV, 100% B; and 16–20 CV, 0% B. The flow rate was set at 2.0 mL/min and the detection wavelength was 280 nm. RA and its two hydrolysis products, DSS and CA (final concentration: ~100 μg/mL), were used as standard samples.

The stability of RA (1 mg/mL) in gel filtration buffer (20 mM Tris–HCl, pH 8.0, and 100 mM NaCl) at 20°C was assessed in the presence and absence of VmFbpA (molar ratio: 1 to 1) by the same RPC analysis. Sampling was performed every week and diluted 100 times with buffer before injecting into a RESOURCE RPC column. The other conditions are the same as described above.

### 5.6 ITC analysis

The 6× His‐free apo VmFbpA samples were dialyzed overnight at 4°C against dialysis buffer (20 mM pH 8.0 HEPES, 50 mM NaCl). ITC was performed using MicroCal iTC200 (Malvern). RA, DSS, or CA was dissolved in dialysis buffer at a final concentration of 400 μM and injected 20 times by a motor‐driven syringe into 200 μL of 20 μM Apo VmFbpA. The first injection volume was 0.1 μL and subsequent injection volumes were 2.0 μL. The reference cell of the microcalorimeter was filled with 200 μL of the corresponding dialysis buffer. The titrations were performed at room temperature. The data were analyzed using MicroCal LLC iTC 200 for Windows (Malvern). All experiments were repeated at least twice to ensure reproducibility.

### 5.7 Comparison of RA, DSS and CA on Fe^3+^ reduction

Solutions containing 40 μM FeCl_3_ and varying concentrations of RA, DSS, and CA (0, 0.625, 1.25, 5, 10, 20, and 40 μM) were prepared and analyzed. Each sample was combined with 0.25% o‐phenanthroline (2 mL) followed by a pH adjustment to 3.5 with HCl, and Milli‐Q water was added to reach a final volume of 25 mL. After incubation at room temperature for 1 h, the absorbance at 510 nm was measured using a microplate spectrophotometer (Benchmark Plus, Bio‐Rad), and the concentration of Fe^2+^ was determined using FeSO_4_·7H_2_O as a standard. The Fe^2+^ concentrations were plotted and fitted as a function of Log_2_(inhibitor concentration) using GraphPad Prism (version: 9.5.0) software with a nonlinear regression model. The half maximal effective concentration (EC_50_) was calculated by reading the inhibitor concentrations at an Fe^2+^ concentration of 20 μM.

### 5.8 6×His‐based VmFbpA inhibition assay (pull‐down assay)

Fe^3+^‐bound His_6_‐VmFbpA (30 μM, 200 μL) was loaded onto a 40 μL Ni‐NTA slurry resin (bed volume: 20 μL) in a Micro Bio‐Spin column (Bio‐Rad) and incubated at 4°C for 30 min. The column was extensively washed with wash buffer (20 mM Tris–HCl, pH 8.0, 50 mM NaHCO_3_, 100 mM NaCl). Various concentrations of RA, DSS, or CA (200 μL) were added to the column and incubated for 60 min at 4°C in a solvent consisting of 20 mM Tris–HCl, pH 8.0, 50 mM NaHCO_3_, and 100 mM NaCl. The column was washed extensively with wash buffer and eluted with 100 μL of elution buffer (wash buffer containing 250 mM imidazole). The Fe concentration of the eluent was determined using ICP‐MS as reported previously (Lu et al. 2019) and adjusted to the protein concentration, which was measured using a Pierce 660 nm Protein Assay. Samples without FeCl_3_ were used as a negative control group and samples lacking inhibitors were used as a positive control group. All buffers contained 0.3% DMSO to solubilize the inhibitors, unless otherwise indicated.

The protein inhibitory rates were calculated using the following formula:

$$\text{Inhibitory rates}\left(\%\right)=\begin{array}{l} \left[1-\frac{\left(\text{Conc.}\;{\mathrm{Fe}}_{\text{sample}}-\text{Conc.}\;{\mathrm{Fe}}_{\mathrm{apo}}\right)}{\text{Conc.}\;{\mathrm{Fe}}_{\text{holo}}-\text{Conc.}\;{\mathrm{Fe}}_{\mathrm{apo}}}\right]\\ \times 100\% \end{array}$$

The inhibitory rate was plotted and fitted as a function of inhibitor concentration by a logarithmic function model using GraphPad Prism (version: 9.5.0) software. The 50% inhibitory concentration (IC_50_) was calculated by reading the concentration at the inhibitory rate of 50%.

### 5.9 Bacteriostatic assay

A pre‐culture of *V. metschnikovii* was prepared in 5 mL of NB medium (3.0 g/L meat extract, 5.0 g/L meat peptone, 10.0 g/L NaCl, pH 7.0) at 37°C. When the OD_600_ reached ~0.6, 2 μL of the pre‐culture was inoculated into 200 μL of NB medium for the bacteriostatic assay. The culture was performed at 37°C in a 96‐well plate. Bacteriostatic agents (RA, DSS, and CA) were tested at a range of concentrations (5000 μM to 39 μM). The cultures without bacteriostatic agents were used as a natural growth control and the cultures without bacteria were used as the respective sterile controls (Blanks). The OD_600_ was measured using a microplate spectrophotometer (Benchmark Plus, Bio‐Rad).

The antibacterial inhibitory rates were calculated based on the following formula after culture for 20 h:

$$\text{Inhibitory rates}\left(\%\right)=\begin{array}{l} \left[1-\frac{\left({\mathrm{OD}}_{600,\mathrm{NG}}-{\mathrm{OD}}_{600,\mathrm{BKs}}\right)-\left({\mathrm{OD}}_{600,\text{Is}}-{\mathrm{OD}}_{600,\mathrm{BKs}}\right)}{{\mathrm{OD}}_{600,\mathrm{NG}}-{\mathrm{OD}}_{600,\mathrm{BKs}}}\right]\\ \times 100\%, \end{array}$$

where NG, Is, and BKs refer to the natural growth control group, experimental groups with inhibitors, and their respective sterile control groups, respectively.

The inhibitory rate was plotted and fitted as a function of Log2(inhibitor concentration) using GraphPad Prism (version: 9.5.0) software with a nonlinear regression model. The MIC was calculated by reading the concentrations at the inhibitory rate of 95%.

## AUTHOR CONTRIBUTIONS

**Koji Nagata:** Conceptualization; supervision; writing – original draft; writing – review and editing. **Peng Lu:** Conceptualization; data curation; investigation; visualization; writing – original draft; supervision; writing – review and editing. **Jinyan Jiang:** Data curation; investigation; writing – original draft. **Chang Liu:** Investigation. **Suguru Okuda:** Writing – original draft. **Hideaki Itoh:** Writing – original draft. **Ken Okamoto:** Writing – original draft. **Michio Suzuki:** Writing – original draft.

## CONFLICT OF INTEREST STATEMENT

The authors declare no conflict of interest.

## Supporting information


**TABLE S1.** X‐ray data collection statistics.
**TABLE S2.** Summary of the refinement statistics.
**FIGURE S1.** Sample preparation of VmFbpA saturated with RA (a) and VmFbpA saturated with Fe^3+^ (b).
**FIGURE S2.** Crystallization and x‐ray diffraction experiments of VmFbpA saturated with RA (a) and VmFbpA saturated with Fe^3+^ (b).
**FIGURE S3.** 2F_O_‐F_C_ map for Fe^3+^‐bound VmFbpA at 1σ (a), 2σ (b), 3σ (c), and 4σ (d). F_O_‐F_C_ map for DSS‐bound VmFbpA at 1σ (e), 2σ (f), 3σ (g), and 4σ (h) (R factor: 0.172). F_O_‐F_C_ map for CA‐bound VmFbpA at 1σ (i), 2σ (j), 3σ (k), and 4σ (l) (R factor: 0.175). The 2F_O_‐F_C_ maps were colored gray. The positive values in F_O_‐F_C_ maps were colored green. No negative values in F_O_‐F_C_ map were observed.
**FIGURE S4.** Chemical structures of RA, DSS, and CA. CA and DSS are the hydrolysis products of RA.
**FIGURE S5.** Fe^2+^ concentrations produced upon the addition of different RA, DSS, and CA concentrations in a 40‐μM Fe^3+^ reaction system (a). Remaining concentration of iron ions after pull‐down experiments upon the addition of different RA, DSS, and CA concentrations to Fe^3+^‐bound VmFbpA solution (b). All experiments were performed in triplicate to confirm the reproducibility.
**FIGURE S6.** Growth curves of *Vibrio metschnikovii* treated with different RA, DSS, and CA concentrations. All experiments were performed in triplicate to confirm the reproducibility.
