# Supplementary material for: Molecular mechanism of Fe3+ binding inhibition to Vibrio metschnikovii ferric ion‐binding protein, FbpA, by rosmarinic acid and its hydrolysate, danshensu
Source: Protein Sci. 2024 Feb 1;33(2):e4881. doi: 10.1002/pro.4881 (PMC10804667; doi:10.1002/pro.4881)
Supplement: Supplementary file 1 — TABLE S1. X‐ray data collection statistics. TABLE S2. Summary of the refinement statistics. FIGURE S1. Sample preparation of VmFbpA saturated with RA (a) and VmFbpA saturated with Fe3+ (b). FIGURE S2. Crystallization and x‐ray diffraction experiments of VmFbpA saturated with RA (a) and VmFbpA saturated with Fe3+ (b). FIGURE S3. 2FO‐FC map for Fe3+‐bound VmFbpA at 1σ (a), 2σ (b), 3σ (c), and 4σ (d). FO‐FC map for DSS‐bound VmFbpA at 1σ (e), 2σ (f), 3σ (g), and 4σ (h) (R factor: 0.172). FO‐FC map for CA‐bound VmFbpA at 1σ (i), 2σ (j), 3σ (k), and 4σ (l) (R factor: 0.175). The 2FO‐FC maps were colored gray. The positive values in FO‐FC maps were colored green. No negative values in FO‐FC map were observed. FIGURE S4. Chemical structures of RA, DSS, and CA. CA and DSS are the hydrolysis products of RA. FIGURE S5. Fe2+ concentrations produced upon the addition of different RA, DSS, and CA concentrations in a 40‐μM Fe3+ reaction system (a). Remaining concentration of iron ions after pull‐down experiments upon the addition of different RA, DSS, and CA concentrations to Fe3+‐bound VmFbpA solution (b). All experiments were performed in triplicate to confirm the reproducibility. FIGURE S6. Growth curves of Vibrio metschnikovii treated with different RA, DSS, and CA concentrations. All experiments were performed in triplicate to confirm the reproducibility. [file PRO-33-e4881-s001.docx]

**Supplementary materials for:**

**Molecular Mechanism of Fe^3+^ Binding Inhibition to *Vibrio metschnikovii* Ferric Ion-Binding Protein, FbpA, by Rosmarinic Acid and its Hydrolysate, Danshensu**

Peng Lu^1†^*, Jinyan Jiang^1†^, Chang Liu^1^, Suguru Okuda^1^, Hideaki Itoh^1^, Ken Okamoto^1^, Michio Suzuki^1^, and Koji Nagata^1,2,3^*

^1^ Department of Applied Biological Chemistry, Graduate School of Agricultural and Life Science, The University of Tokyo, 1-1-1 Yayoi, Bunkyo-ku, Tokyo 113-8657, Japan

^2^ Agricultural Bioinformatics Research Unit, Graduate School of Agricultural and Life Science, The University of Tokyo, 1-1-1 Yayoi, Bunkyo-ku, Tokyo 113-8657, Japan

^3^ Research Center for Food Safety, Graduate School of Agricultural and Life Science, The University of Tokyo, 1-1-1 Yayoi, Bunkyo-ku, Tokyo 113-8657, Japan

^†^ These authors contributed equally to this work.

* Corresponding author.

E-mail: porterlu@g.ecc.u-tokyo.ac.jp (P. Lu)

E-mail: aknagata@mail.ecc.u-tokyo.ac.jp (K. Nagata)

# Appendix A. Supplementary data

The following are the Supplementary data to this article:

**Supplementary Table 1**.

X-ray data collection statistics.

**Supplementary Table 2**.

Summary of the refinement statistics.

**Supplementary Figure 1**.

Sample preparation of VmFbpA saturated with RA (**A**) and VmFbpA saturated with Fe^3+^ (**B**).

**Supplementary Figure 2**.

Crystallization and X-Ray diffraction experiments of VmFbpA saturated with RA (**A**) and VmFbpA saturated with Fe^3+^ (**B**).

**Supplementary Figure 3.**

**A:** 2F_O_-F_C_ map for Fe^3+^ bound VmFbpA at 4σ. **B:** F_O_-F_C_ map for DSS bound VmFbpA (R factor: 0.172). **C:** F_O_-F_C_ map for CA bound VmFbpA (R factor: 0.175)

**Supplementary Figure 4**.

Chemical structures of RA, DSS, and CA. CA and DSS are the hydrolysis products of RA.

**Supplementary Figure 5**.

**A**: The concentrations of Fe^2+^ produced upon addition of different concentrations of RA, DSS, and CA in a 40 μM Fe^3+^ reaction system. **B**: The remaining concentration of iron ions after pull-down experiments upon addition of different concentrations of RA, DSS, and CA to Fe^3+^ bound VmFbpA solution. All experiments were performed in triplicate to confirm the reproducibility.

**Supplementary Figure 6**.

Growth curves of *Vibrio metschnikovii* treated with different concentrations of RA, DSS, and CA. All experiments were performed in triplicate to confirm the reproducibility.

**Supplementary Table 1** X-ray data collection statistics*

| Data collection | Fe^3+^-bound VmFbpA | RA-treated VmFbpA |
| --- | --- | --- |
| PDB ID | 8J4J | 8J4H |
| Beamline | SPring-8 BL44XU | PF-AR NE3A |
| Detector | EIGER X16M | PILATUS 2M-F |
| Wavelength (Å) | 0.899995 | 1.00000 |
| Space group | *P*6_3_22 | *P*6_3_22 |
| Resolution (Å) | 43.781 - 2.153  (2.164 - 2.153) | 49.825 - 2.008  (2.018 - 2.008) |
| Unit cell parameters |  |  |
| a, c (Å) | 91.52, 150.45 | 90.23, 149.47 |
| Total  reflections | 390,346 (15,402) | 478,925 (73,284) |
| Unique  reflections | 38,034 (1,457) | 45,218 (7,187) |
| Completeness (%) | 99.5 (97.4) | 99.3 (97.9) |
| Redundancy | 10.26 (9.70) | 10.59 (10.20) |
| Rmerge (%) | 10.3 (176.7) | 13.2 (206.0) |
| Rpim (%) | 3.7 (81.6) | 3.4 (71.3) |
| CC 1/2 (%) | 99.9 (78.9) | 99.9 (70.0) |
| <I / σ (I)> | 11.02 (1.19) | 15.81 (3.92) |

*The values in parentheses represent the data for highest resolution shell.

**Supplementary Table 2** Summary of the refinement statistics

| Refinement parameters | Fe^3+^-bound VmFbpA | RA-treated VmFbpA |
| --- | --- | --- |
| Rwork / Rfree | 0.183 / 0.246 | 0.172 / 0.212 |
| No. of atoms |  |  |
| Protein | 2,364 | 2,364 |
| Ligand/Ion | 9 (HCO_3_^-^, Fe^3+^) | 14 (DSS) |
| Water | 116 | 231 |
| r.m.s. deviations | | |
| Bond lengths (Å) | 0.0069 | 0.0079 |
| Bond angles (º) | 1.5017 | 1.5392 |
| Ramachandran plot | | |
| Favored (%) | 97.72 | 97.72 |
| Allowed (%) | 2.28 | 2.28 |
| Outliers (%) | 0.00 | 0.00 |
| MolProbity score | 1.55 | 1.87 |


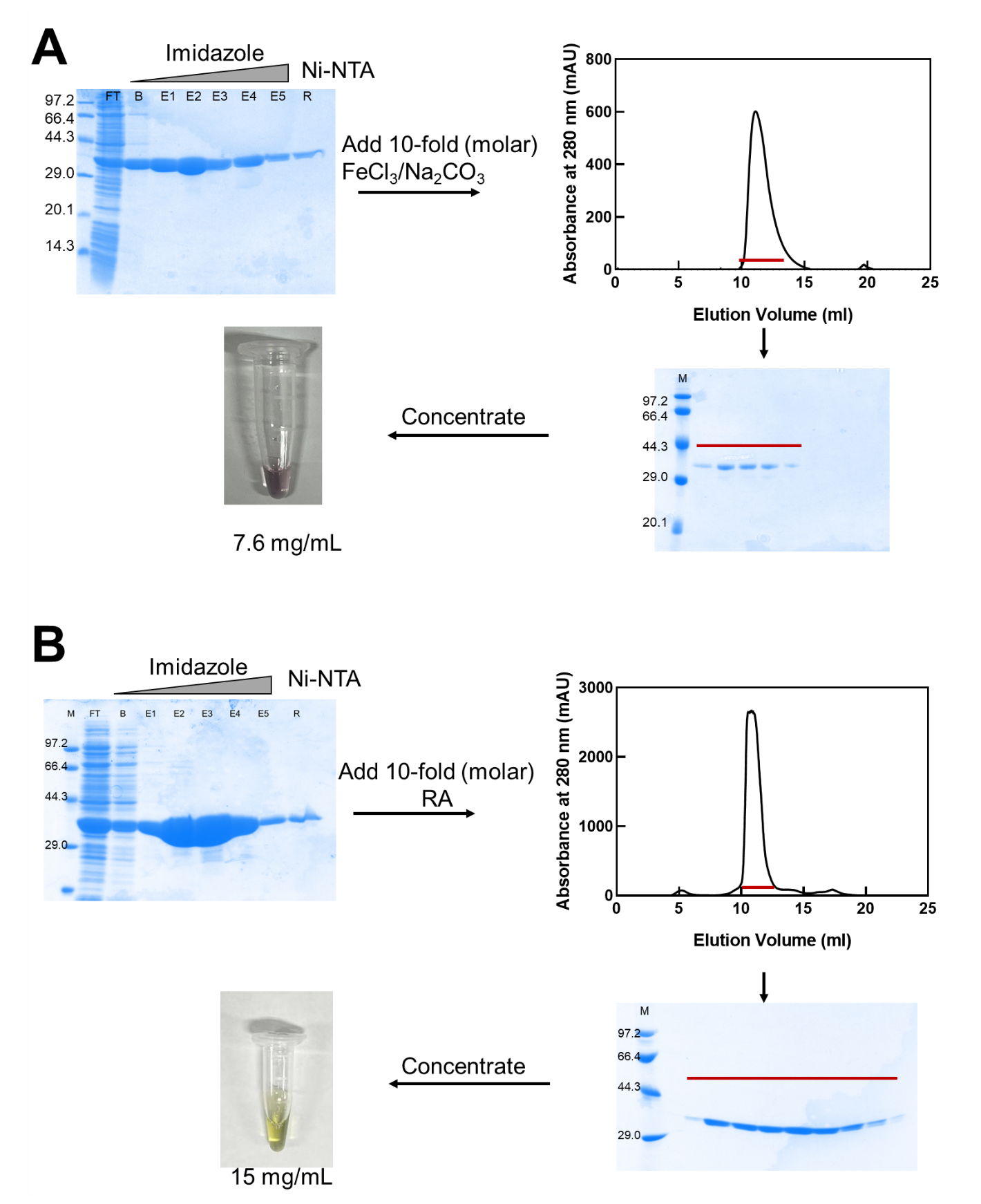


Supplementary Figure 1 Lu. *et al.*

*
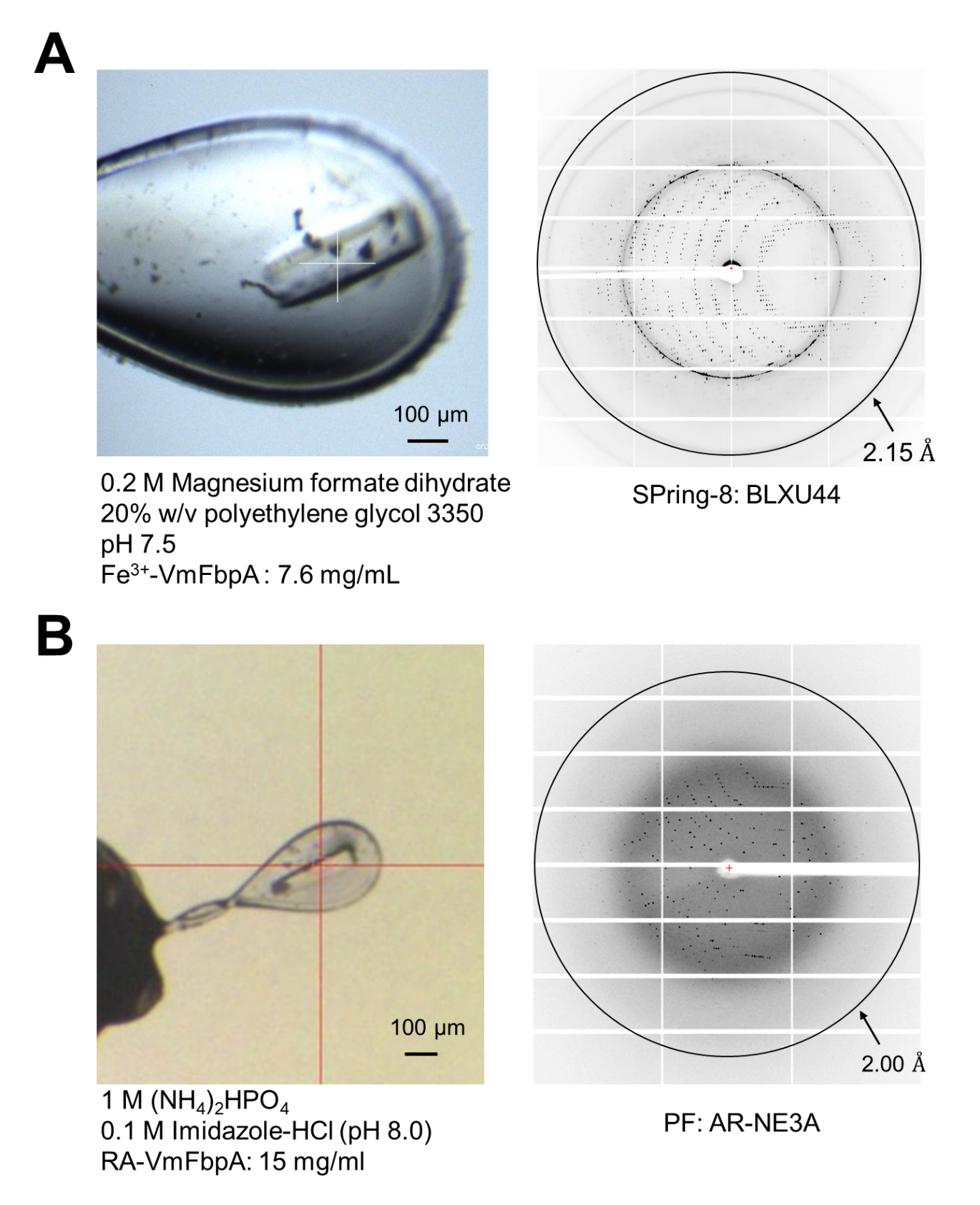
*

Supplementary Figure 2 Lu. *et al.*


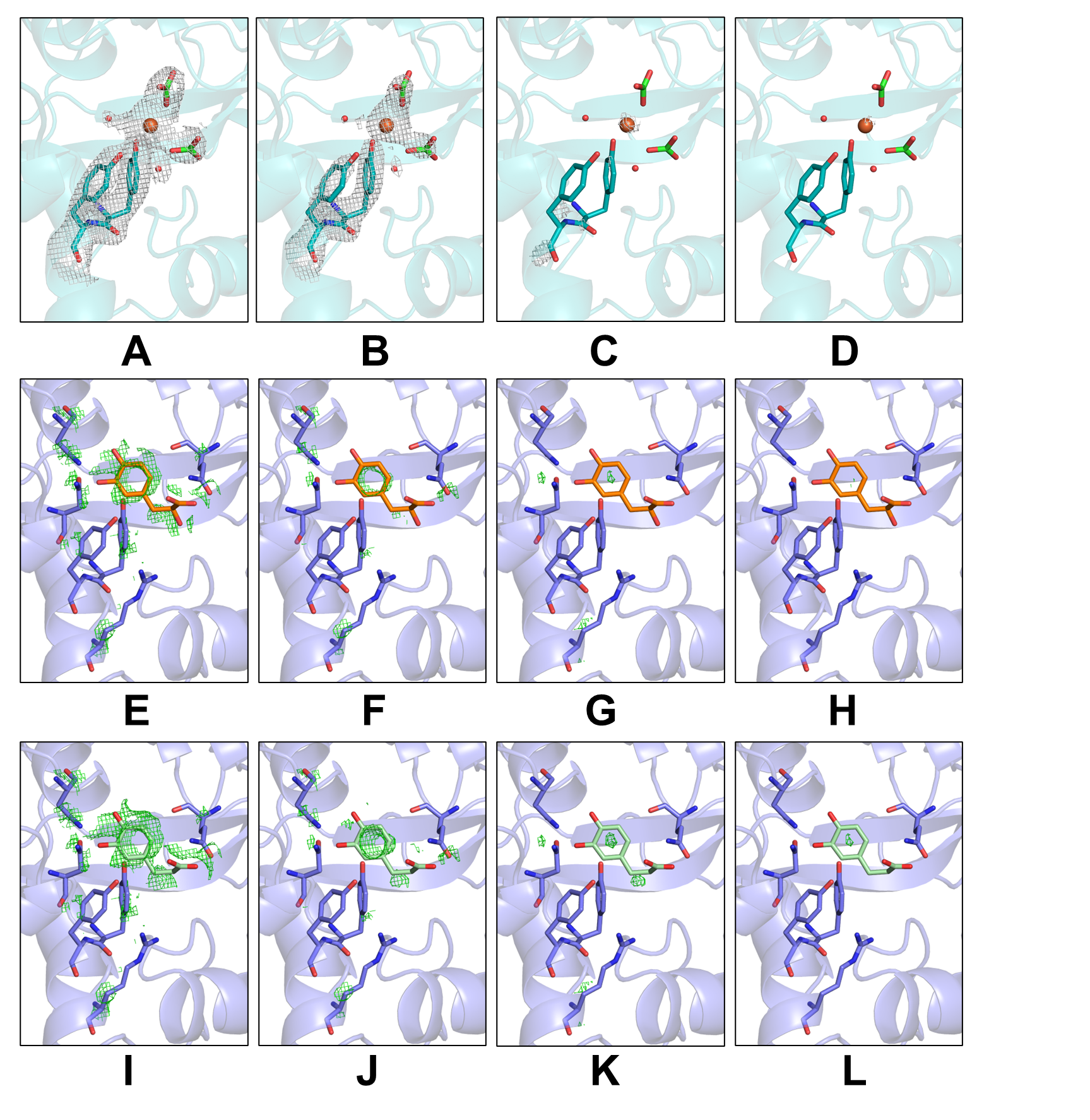


Supplementary Figure 3 Lu. *et al.*


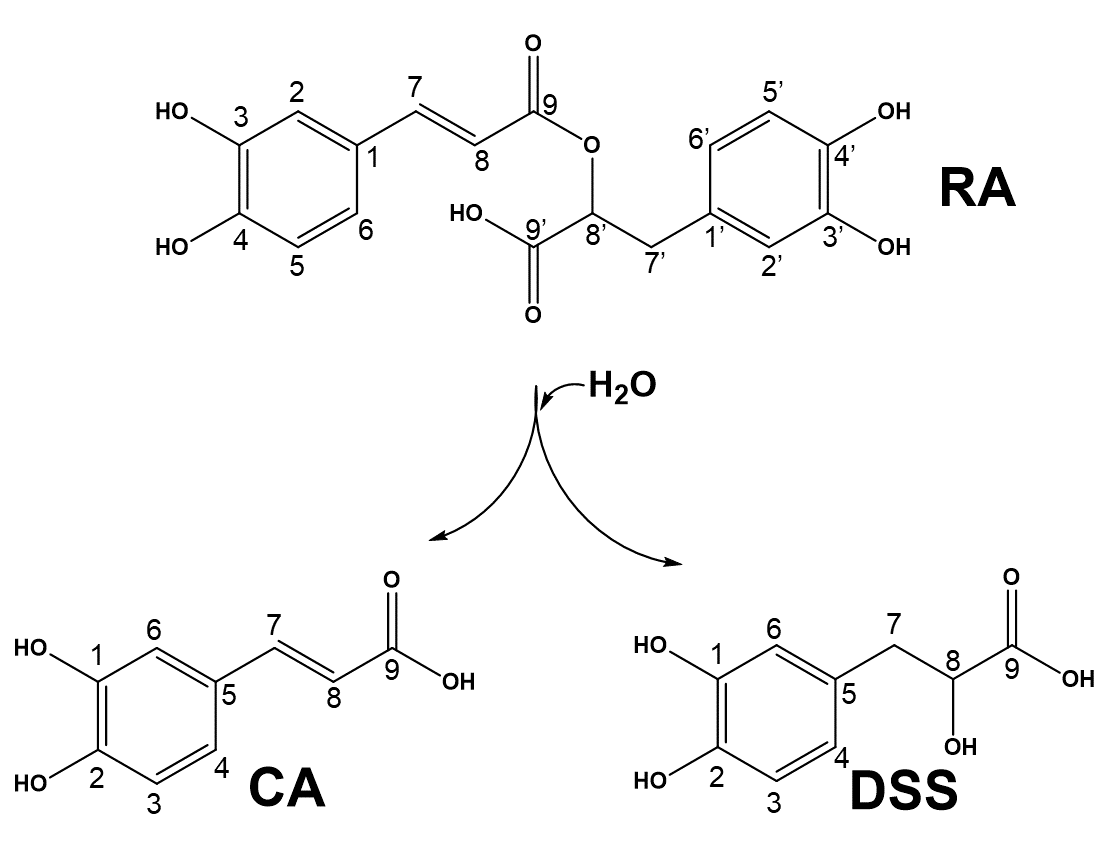


Supplementary Figure 4 Lu. *et al.*

*
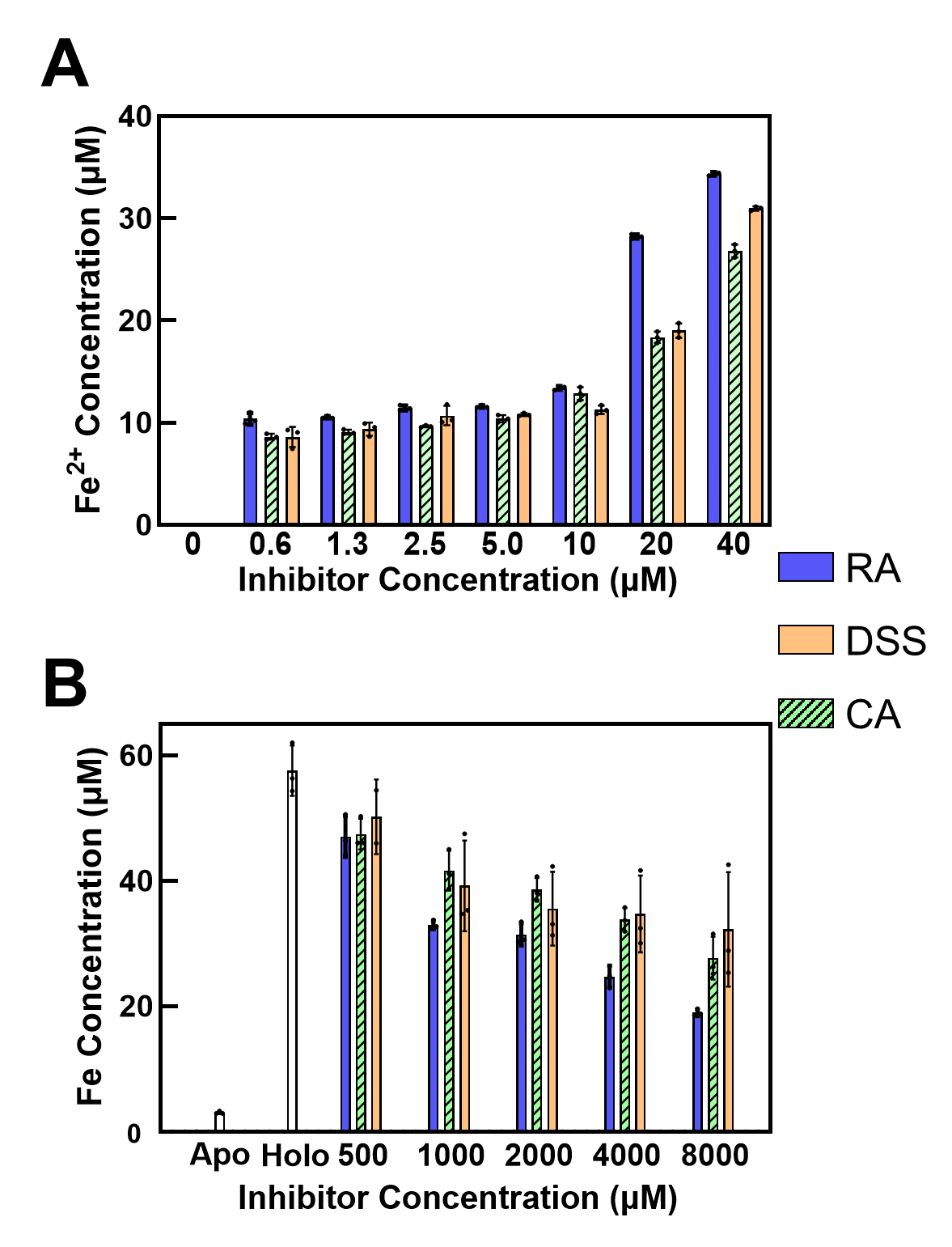
*

Supplementary Figure 5 Lu. *et al.*


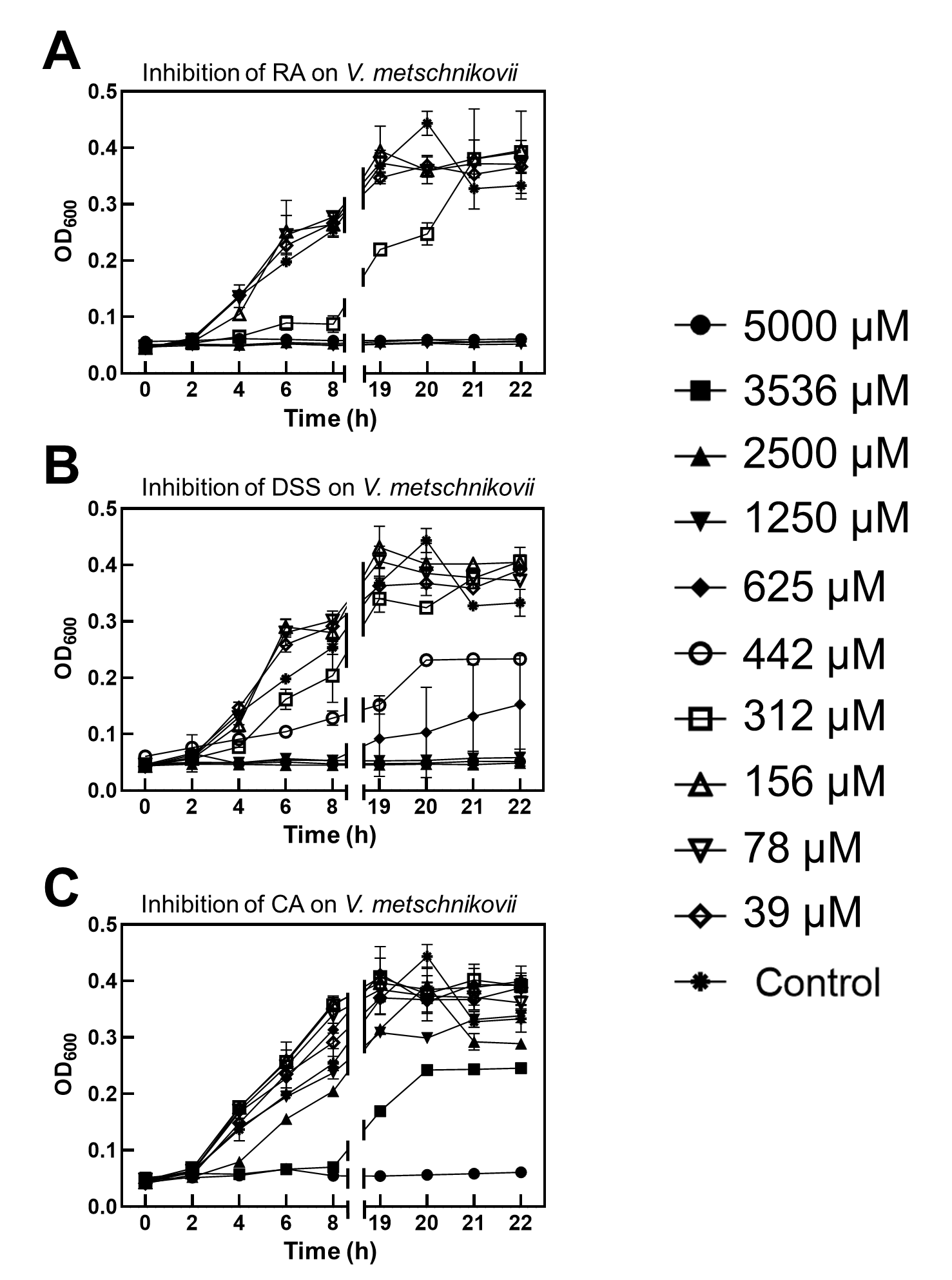


Supplementary Figure 6 Lu. *et al.*
